# Early life growth is related to pubertal growth and adult height – a *QEPS*-model analysis

**DOI:** 10.1038/s41390-025-03939-9

**Published:** 2025-02-25

**Authors:** Carin Skogastierna, Anton Holmgren, Aimon Niklasson, Andreas F. M. Nierop, Aldina Pivodic, Anders Elfvin, Diana Swolin-Eide, Kerstin Albertsson-Wikland

**Affiliations:** 1 Department of Pediatrics, Institution of Clinical Sciences, Sahlgrenska Academy, University of Gothenburg, Gothenburg, Sweden; 2 Region Västra Götaland, Department of Pediatrics, The Queen Silvia Children’s Hospital, Sahlgrenska University Hospital, Gothenburg, Sweden; 3 Department of Pediatrics, Halland Hospital, Halmstad, Sweden; 4 Department of Research and Development, Region Halland, Sweden; 5 Muvara bv, Multivariate Analysis of Research Data, Leiderdorp, The Netherlands; 6 Department of Physiology/Endocrinology, Institute of Neuroscience and Physiology, Sahlgrenska Academy, University of Gothenburg, Gothenburg, Sweden; 7 APNC Sweden, Mölndal, Sweden; 8 Department of Ophthalmology, Sahlgrenska University Hospital, Västra Götaland Region, Gothenburg, Sweden

## Abstract

**Background:**

The early life growth period, from conception to ~2 years of age, has proven crucial for later health. We hypothesized that early life growth could explain variations in pubertal growth and timing, and adult height.

**Methods:**

This retrospective, population-based study was conducted in Sweden. A subgroup, including individuals of all gestational ages and birth sizes (*n* = 4700, 50% males), from the longitudinal GrowUp_1974&1990_Gothenburg cohorts was used. *QEPS* variables were analyzed in univariate and multivariate linear regression models, separately per sex; *Q*-function throughout all growth periods, and specific *E*- and *P*-functions, for early life growth and pubertal growth, respectively.

**Results:**

In multivariate models, early life growth explained 37–38% of the variability in specific pubertal growth, but less so the variability in pubertal timing. Variability in adult height was explained by birth size (57–62%), early growth (66–67%), childhood growth (65–69%), and to a lesser degree by mid-parental height (35–39%). The change in height during puberty explained 8–9% of the variation in adult height.

**Conclusion:**

This study indicates that early life growth is strongly associated with the variability in pubertal growth, and adult height, but not with the timing of pubertal growth.

**Impact:**

- Early life growth is important as it can serve as a marker for future growth, development, and health.
- The association between length growth during fetal life and infancy and pubertal growth and timing, and adult height, is only partly understood.
- Using the *QEPS* growth model, specific early life growth (*E*-function) and specific pubertal growth (*P-*function), including individual variations in tempo and amplitude, can be studied separately from ongoing basic growth (*Q-*function).
- This study showed that early life growth is strongly associated with and explains specific pubertal height gain and adult height but less so the timing of pubertal growth.

## Introduction

Growth during early life reflects the current health of the forming individual and might be used as a marker for future growth, development, and health.^1^ The early life growth period, from conception until the transition between infancy and childhood at approximately 2 years of age, has been proven crucial for morbidity and mortality risks in adult life, and may also be important for growth later in life.^1–3^

Body growth is the cumulative sum of cell proliferation and cell enlargement that occurs in multiple tissues, whereas longitudinal bone growth, referred to as length growth for infants, and height growth thereafter, takes place at the growth plate.^4^ Early life growth research has mainly centered on weight due to its widespread data availability, globally. Nevertheless, are changes in weight an unsensitive marker of growth and do not differentiate lean mass from fat mass.^5^ This work focuses on length/height growth.

A child’s growth can be divided into different phases: early life (fetal–infancy), pre-pubertal childhood, and puberty,^6^ each governed by unique regulatory mechanisms. Human growth is a multidimensional process. The increase in size over time shapes the individual’s growth trajectory, meanwhile the parallel process of maturation (acquisition of adult features) is ongoing.^7^ The fetal growth period is characterized by formation of organs and sex differentiation, followed by rapid body growth. Regulation of fetal growth involves interactions between the mother, placenta, and fetus, with maternal height being a major determinant of fetal size.^8^

Growth in length during infancy is mainly regulated by nutrition.^4^ Hormones in breast milk, like leptin and ghrelin, are likely contributing to the metabolic programming, thus regulating infant growth.^9^ Length growth is highly sensitive to external disturbances during infancy but loses much of its vulnerability at about 2 years of age, when the childhood growth period starts, mainly driven by growth hormone.^4,10^ The pubertal growth spurt, induced by the re-activation of the hypothalamic–pituitary–gonadal axis and driven by estrogen, is unique to humans.^11^ The timing of puberty is partly inherited,^12^ but the ongoing secular trend of declining age at pubertal onset^13–15^ has highlighted the importance of environmental influences.^16^

Early life experiences may influence pubertal timing. Nutritional status in early life is a key regulator of early growth and is also considered an important modulating factor for pubertal development.^17^ Rapid weight gain during infancy and early childhood has been associated with an earlier onset of puberty,^18,19^ particularly in females with a low birthweight.^20,21^ While some studies also link early length gains to an earlier onset of puberty, it remains uncertain to what extent these results can be attributed to the corresponding weight gain.^22,23^ The associations between early life growth and the magnitude of pubertal growth remain mostly unexplored,^24^ but earlier studies of the GrowUp 1974 Gothenburg cohort,^25,26^ showed that greater length at birth and in infancy, were associated with more height gain during puberty.^24^

Chronic stress in early life is believed not only to impair growth but also to predispose towards an earlier onset of puberty,^27,28^ likely through premature activation of neuroendocrine axes in early infancy.^28^ Notably, premature infants and those born SGA (small for gestational age) have been shown to exhibit heightened hormonal activity during the first months of life, potentially predisposing them to altered pubertal trajectories.^29,30^ Yet, the published evidence does not suggest that being born preterm leads to a significant acceleration in the onset of puberty.^31,32^

In late puberty, high estrogen levels cause fusion of the epiphysis, resulting in the attainment of adult height.^4^ Both pubertal growth^33–35^ and adult height^36^ are assumed to be strictly genetically regulated. It has been found that independent loci signals can influence either basic growth from birth to adulthood or specific pubertal growth.^35^ Auxological status at birth has been shown to be strongly correlated with attained adult height.^37^ However, an individual’s growth trajectory can be perturbated by environmental factors, resulting in an impaired adult height.^38^ Consequently, attained adult height might serve as an accurate indicator of early life conditions, particularly nutrition.^39^ Postnatal overnutrition can lead to rapid length/height growth but likely does not increase adult height, while early malnutrition can permanently impact final adult height.^4^ As shown in an earlier study of the GrowUp 1974 Gothenburg cohort,^40^ adult short stature is commonly a result of suboptimal growth during multiple phases, with growth failure prior to 12 months of age being the most critical.^40^ Additionally, growth phases seem to be interrelated; height gain in one phase can influence the magnitude of height gain in subsequent phases.^40^

A growth model enables detailed study of growth periods, including exploration of their regulation. The *QEPS* (Quadratic-Exponential-Pubertal-Stop) -model^41^ is one of the two growth models^6,41^ describing growth from fetal life to adulthood, allowing studies of early life growth. The *QEPS*-model also quantifies the tempo and amplitude of early life (*E*-function) and pubertal (*P*-function) growth. Moreover, the *QEPS*-model enables detail investigation of specific early life (*E*-function) and specific pubertal (*P*-function) growth, which can be separated from basic ongoing (*Q*-function) growth.^41^ By describing longitudinal length/height data with the *QEPS* growth model, our goal is to contribute to a deeper understanding of early life growth dynamics and their implications for future growth.

The aim of the present study was to explore how growth during early life relates to pubertal growth and timing, and how growth during different growth periods explain variations in adult height. We hypothesized that early life growth is related to both the timing and amplitude of pubertal growth, as well as the attained adult height.

## Material and methods

### Study population

The Combo GrowUp 1974/1990 cohort consisted of 6769 (50% males) individuals from the GrowUp 1974^25,26^ and GrowUp 1990 Gothenburg cohorts with individuals of all gestational ages and birth sizes.^26,42^ Included in this cohort were individuals with longitudinal height measurements at all age periods^6^ (infancy, childhood, puberty, and adult height) and adult height measured by the research team at 18–19 years of age. Information on gestational age, birth length, and birth weight was also available in the Swedish medical birth registry for all individuals. Of the 6769 participants, 3057 (49% males) were born in 1974 and 3712 (51% males) were born in 1990. Longitudinal growth data were collected from well-baby clinics, and records from child healthcare centers and school healthcare.

A subgroup of 4700 participants (50% males) from the Combo GrowUp 1974/1990 cohort who met the inclusion criteria were included in this study. Only individuals not affected by chronic diseases (except for asthma/allergy without medication),^26^ and for whom height data from both parents were known, were included. All children were of Nordic ethnicity^25^ (1974 cohort) or had at least one Nordic parent^42^ (1990 cohort). Supplementary Fig. 1 shows drop-out numbers. A non-participant drop-out analysis was performed using the 993 individuals fulfilling all inclusion criteria except for known height of both parents and is presented in Supplemental Table 1. Of the 4700 participants, 2201 (48% male) were born in 1974, and 2499 (52% male) were born in 1990.

For participants born in 1974, the birth weights were ranging from 1530 g to 5420 g (mean 3524 g) among males and from 1130 g to 5670 g (mean 3395) among females. For participants born in 1990, the birth weighs were ranging from 785 g to 5310 g (mean 3595 g) among males and from 1045 g to 5430 g (mean 3501 g) among females. In 1974, 1810 participants (82%) were born at term (37 + 0 to 42 + 0 weeks/days of gestation), 315 (14%) post-term (>42 + 0 weeks/days of gestation) and 76 (3%) preterm (<37 + 0 weeks/days of gestation), of which 2 were born very preterm (28 + 0 to 32 + 0 weeks/days of gestation) and 0 extremely preterm (<28 + 0 weeks/days of gestation). The figures for the participants born in 1990 were 2198 (88%) term, 181 (7%) post-term, 105 (4%) preterm, 14 very preterm, and one extremely preterm.

### Study design

**Fig. 1:**
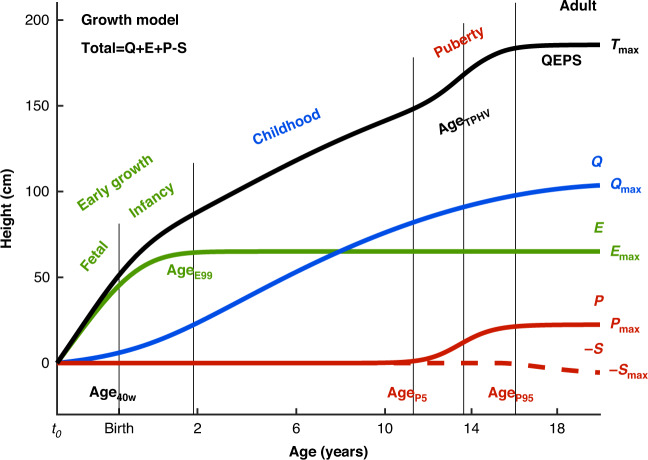
Design of the study. The total height (*QEPS*) is the sum of four growth functions: a quadratic growth function (*Q*), a negative exponential growth function (*E*), a pubertal growth function (*P*) and a stop function (*S*) modeling the end of growth for function *Q*. B = birth, t_0_ = about 6 weeks after conception. Birth is marked with a vertical line. Age scale below 3 years is stretched out. The growth periods (early life: fetal-infancy, childhood, and puberty) are defined by the QEPS estimates *Age*_*40w*_, *Age*_*E99*_, *Age*_*P5*_, *Age*_*TPHV*_, and *Age*_*P95*_. The contribution of each growth function (*Q*_*max*_*, E*_*max*_*, P*_*max*_, and *S*_*max*_) to the total height, *T*_*max*_, is illustrated to the right.

Individual, longitudinal length/height curves from birth to adult height were obtained using the longitudinal height measurements and the growth functions of the *QEPS*-model.^41^ Analyses were based on the *QEPS*-model derived for each growth period [early life (fetal–infancy, including birth), childhood, and puberty] (Fig. 1). The importance of these growth periods for the studied outcomes were assessed in univariate and multivariate linear regression models, separately per sex.

### The QEPS growth model

The *QEPS* growth model^41^ was used to describe individual length/height growth from birth to adult height, defining an individual’s growth as a combination of four distinct mathematical functions; a Quadratic *(Q)* function, for the basic ongoing growth starting from early fetal life until the end of growth, stopped by a *Stop (S*) function, a negative Exponential (*E*) function, specific for growth during fetal life and infancy, and a non-linear Pubertal (*P*) function specific for pubertal growth (Fig. 1). The total height (*T*) in cm is a function of age: *T*(age) = *Q*(age) + *E*(age) + *P*(age) − *S*(age).^41^ The total height when growth is finished is accordingly defined as: *T*_*max*_ *=* *Q*_*max*_ *+* *E*_*max*_ *+* *P*_*max*_ *−* *S*_*max*_.

The different growth functions can be modified for *E* and *P* by both time-scale (*E*_*timescale*_, *P*_*timescale*_) and height-scale parameters (*E*_*heightscale*_*, P*_*heightscale*_), and for *Q* and *S* by height-scale parameters (*Q*_*heightscale*_*, S*_*heightscale*_), thus describing individual growth with a shape-invariant model. All *QEPS* time-scale and height-scale parameters have ratio values with individual *QEPS* values in the numerator and corresponding typical mean function values of the original *QEPS* model in the denominator. *E*_*timescale*_ modifies the time scale of the *E*-function growth, and is therefore inversely related to the tempo of *E*. The height-scale parameters, i.e. the individual height-scale ratios, modifies the height scale of the *Q*, *E, P*, and *S*- functions growth.

Separate curve fitting for each individual gives six parameter estimates with CI (confidence interval) and the possibility to compute many derived variables and individual *QEPS*-function values at all ages.

For all *QEPS* variables (except *E*_*timescale*_), the height and the individual’s current age were given in cm and in weeks/months/years. The SDS (standard deviation score) for the *QEPS* variables were computed from the reference population presented by Albertsson-Wikland et al. in 2020.^42^ Validated externally,^43^ the *QEPS*-model also incorporates measures and tools such as CI and Mathselect to ensure data reliability.^41^

### Outcome variables

The primary outcome variable was specific pubertal growth (*P*_*max*_*SDS*), i.e. the total height gain related to the *P-*function in SDS_._ Secondary outcomes were the timing of pubertal growth, evaluated by age at pubertal onset, specifically age at which 5% of the *P*-function growth is reached (*Age*_*P5*_), and age at mid-puberty, i.e. age at PHV (peak height velocity),^44,45^ calculated from the total growth curve (*Age*_*TPHV*_), and adult height in SDS (*AH SDS)*.

### Explanatory variables

#### Parental heights

Mother’s heights were obtained from the Swedish medical birth register, and father’s heights were obtained from a questionnaire answered by parents or study participants, or from records at child healthcare centers.^26^ *Parental height SDSs* were derived from the current study population (*n* = 4700) by computing separately the SDS based on the heights of all fathers and all mothers using the formula (height – mean height)/standard deviation. MPH (mid-parental height) was calculated as the mean of the heights of the mother and father, and was summarized in centimeters and in SDS (*MPH SDS)*.

#### Gestational age and birth size

For both cohorts, data originated from the Swedish medical birth register. Gestational age was estimated based on last menstrual period in the GrowUp Gothenburg 1974 cohort,^25,26^ and based on either ultrasound or timing of last menstrual period in the 1990 cohort.^26,42^ *Birth length SDS* and *birth weight SDS* were derived from a growth reference based on 800,000 healthy Swedish children born between 1990 and 1999.^25^

##### Basic growth and early life growth

Basic, ongoing growth, starting in fetal life and ending in adulthood, was described by *Q-*function and *Q*_*max*_, for the prepubertal period. Specific, early life growth was described by the *E-*function, *E*_*max*_ and *E*_*timescale*_. In the *QEPS*-model, *Q* and *E* are prepubertal functions that are fitted with prepubertal measurements. They are extrapolated to adulthood and can be used for making a prepubertal reference to adulthood.^41^ Therefore, *Q*_*max*_ and *E*_*max*_ should be considered as extrapolated prepubertal function values in adulthood.

##### The fetal–infancy growth period

Fetal growth ends at birth and continues as postnatal growth in infancy. In our study, we investigated the change in length growth between *birth* and age at 40 weeks of gestation (*Age*_*40w*_), which is of importance for individuals born before 40 gestational weeks, as well as after *Age*_*40w*_. The *QEPS* model^41^ allowed us to estimate prenatal growth in participants born at more than 40 gestational weeks. The ending of *the early life (fetal–infancy) growth period* was here defined as the age at which 99% of the *E*-function growth is reached (*Age*_*E99*_), which is individually defined and corresponds approximately to the traditionally used 2-year duration of the infancy growth period.^6^ For comparison, age at which 95% of the *E*-function growth is reached (*Age*_*E95*_) corresponds to an age of around 12 months (data not shown).

##### The childhood growth period

Duration of the childhood growth period: from *Age*_*E99*_ to the onset of the specific pubertal growth period, *Age*_*P5*_.

Childhood height gain: prepubertal growth was calculated versus the parameters *Q*_*max*_*, E*_*max*_ and *QE*_*max*_, based on the prepubertal *Q* and *E* functions of the *QEPS* model (Fig. 1).

##### The pubertal growth period

Timing of pubertal growth: in this study, we defined the onset of the pubertal growth period as the age when 5% of *P*-function growth was attained (*Age*_*P5*_). To simplify comparison with other studies, age at PHV^45,46^ was also calculated from the total growth curve (*Age*_*TPHV*_*)*. *Age*_*P50*_ corresponds to the timepoint when 50% of the P-function growth has been completed.

Duration of the pubertal growth period: from *Age*_*P5*_ to the end of the pubertal growth period, here defined as the age at which 95% of *P*-function growth has been completed (*Age*_*P95*_).

Pubertal growth: during puberty, the *QEPS*-model can separate specific pubertal *P-*function growth, *P*_*max*_ or *P*_*P5-P95*_, from the ongoing basic *QES-*functions of growth (*QES*_*P5-P95*_*)* and thus describe the total gain in height during puberty (*T*_*P5-P95*_*). P*_*max*_ was transformed to SDS using the mean value and standard deviation for the current study population (*P*_*max*_*SDS*).

##### Adult height and growth differences

Total growth (T_max_): The total height (*T*) in centimeters is a function of age: *T*(age) = *Q*(age) + *E*(age) + *P*(age) − *S*(age), which is assumed to have ended when all *QEPS* functions have reached their maximum amplitude (*T*_*max*_ *=* *Q*_*max*_ *+* *E*_*max*_ *+* *P*_*max*_ *−* *S*_*max*_).

Measured adult height (AH_measured_): height was measured at around 18 years of age using a stadiometer and was based on the mean of three measurements. Adult height was defined as having been attained once the increase in height over the previous 12 months was <0.5 cm; individuals still experiencing growth underwent additional measurement until adult height was attained.^26^

Adult height (AH): in the current study used as adult height outcome. The calculated *T*_*max*_ served as basis for this variable. If *AH*_*measured*_ exceeded *T*_*max*_, it was utilized as the designated adult height outcome (*AH*), otherwise *T*_*max*_ was used.^47^ *AH* was transformed to SDS (*AH SDS*).

Diff SDSs: the calculated differences between the individual’s length/height in SDS at *Age*_*26w*_, *birth, Age*_*40w*_, *Age*_*E99*_, *Age*_*P5*_, *Age*_*TPHV*_*, Age*_*P95*_, and *AH* and the individual *MPH SDS* (i.e. the intrafamilial height difference).

Change X_y-z_ SDS: the calculated growth difference in SDS of the actual *QEPS* variable (*X*) between the ages *y* and *z*.

### Data handling and statistical analysis

To construct longitudinal growth curves for each individual in the study population, height data were exported to Matlab®, and *QEPS*-model parameters were automatically estimated by fitting the height data from each individual separately (The MathWorks, Inc., Natick, MA, v. 9.3.0 R2017b). Longitudinal growth curves were individually, visually reviewed in Matlab®. All *QEPS* variables were also reviewed using scatter plots in IBM SPSS® software (IBM SPSS Statistics for Windows, Version 29.0. Armonk, NY: IBM Corp). Statistical analyses were performed using SAS software® version 9.4 (SAS Institute Inc., Cary, NC). A *p*-value < 0.05 was considered statistically significant following adjustment using the Bonferroni–Holm technique.

Continuous variables were described by the mean, standard deviation, median, minimum, maximum, and CI, and categorical variables by counts and percentages.

Associations between explanatory predictors, and outcome variables were performed by first applying univariate linear regression models. Associations between continuous explanatory variables and outcome variables were investigated visually in scatter plots, applying locally estimated scatterplot smoothing to decide whether non-linear models (using either splines, piecewise linear associations, or categories) were required. None of the associations required a non-linear approach.

Then *multivariate linear regression models* were developed separately for the *different growth domains*, i.e. different growth periods, of the explanatory variables to decrease the problem of expected multicollinearity. The variable inflation factor was studied for this purpose. The statistically independent predictors from each growth domain were selected for evaluation in the final *total multivariate model*. Variable selection was defined using both stepwise forward and backward selection, considering the variable inflation factor and the problem of changing effects for variables that are part of a model with other highly correlated variables.

Both predictors and outcome variables were standardized for their own standard deviation in the cohort, by sex, in order to allow comparison of the standardized beta coefficients for the predictors between and within different outcome variables. Standardized beta explains how much an outcome variable is changing in SDs when the predictor is increasing one SD. Additionally, in simple (univariable) linear regression, standardized beta corresponds to Pearson correlation coefficient r that provides an easier interpretation. However, this is not the case in the multivariable models. R^2^ describes the proportion of the variance in the dependent variable that is explained by the independent variables. Standardized beta coefficients from the multivariate models were described in a forest plot along with their 95% CIs.

Additionally, R^2^ and partial R^2^ were presented describing explained variance. Interactions between explanatory variables and sex were also investigated. For completeness, despite redundancy in the results due to a perfect correlation between all *Q*-function variables at different selected timepoints, all variables were presented for the univariate analyses. However, only one of those was selected in the Bonferroni–Holm adjustment and in the multivariate models. Within each subheading of the explanatory variables, Bonferroni–Holm adjustment was applied separately for each outcome in order to keep the Type I error rate low.

For tests between two groups, Fisher’s exact test was used for dichotomous variables, and the Mann–Whitney U-test for continuous variables.

### Ethics

The Grow-Up Gothenburg studies were approved by the Regional Ethics Review Board in Gothenburg, Sweden (Ethics number 91-92, 19 March 1992, number 444-08, 18 August 2008, and updated number 1000-15, 11 January 2016). All participants gave informed written consent; informed consent from legal guardians was also given for individuals <18 years of age.

## Results

All analyses were performed separately for males and females. Descriptive statistics for the total study population (*n* = 4700) are presented in Table 1 (the measured baseline characteristics in Table 1a, and the *QEPS* estimates for the different growth phases in Table 1b).

**Table 1:** a Baseline characteristics of the study population. b *QEPS* estimates for the study population

a			
Variable	Male *N* = 2355	Female *N* = 2345	*p*-value
*Birth characteristics*			
Birth length (cm)	50.6 ± 2.4 51.0 (34.0–60.0) *n* = 2355	49.9 ± 2.3 50.0 (36.0–58.0) *n* = 2345	<0.0001
Birth length (SDS)	−0.65 ± 1.27 −0.64 (−6.00–5.03) *n* = 2355	−0.66 ± 1.26 −0.68 (−5.77–4.39) *n* = 2345	0.44
Birth weight (grams)	3563.4 ± 568.6 3580.0 (785.0–5420.0) *n* = 2355	3448.8 ± 531.2 3450.0 (1045.0–5670.0) *n* = 2345	<0.0001
Birth weight (SDS)	−0.45 ± 1.12 −0.34 (−6.67–2.81) *n* = 2355	−0.43 ± 1.11 −0.37 (−5.72–3.55) *n* = 2345	0.97
Gestational age (weeks)	40.1 ± 1.8 40.5 (28.7–44.5) *n* = 2355	40.2 ± 1.7 40.5 (26.4–45.1) *n* = 2345	0.0056
*Parental heights*			
Father’s height (cm)	180.26 ± 6.79 180.00 (156.00–204.00) *n* = 2355	180.86 ± 6.73 180.00 (155.00–208.00) *n* = 2345	0.0012
Father’s height (SDS)	0.00 ± 1.00 −0.07 (−3.48–3.42) *n* = 2355	−0.00 ± 1.00 −0.03 (−3.91–4.21) *n* = 2345	0.87
Mother’s height (cm)	167.07 ± 5.99 167.00 (148.00–189.00) *n* = 2355	166.76 ± 5.91 167.00 (148.00–188.00) *n* = 2345	0.14
Mother’s height (SDS)	0.00 ± 1.00 0.03 (−3.06–3.61) *n* = 2355	0.00 ± 1.00 −0.04 (−3.10–3.52) *n* = 2345	0.11
Mid-parental height (SDS)	0.00 ± 0.78 −0.02 (−2.59–2.81) *n* = 2355	−0.00 ± 0.79 −0.01 (−3.42–2.73) *n* = 2345	0.76
*Adult height*			
Adult height (cm)	181.32 ± 6.66 181.13 (157.29–203.93) *n* = 2355	168.14 ± 6.21 168.10 (146.50–190.50) *n* = 2345	<0.0001
Adult height (SDS)	0.00 ± 1.00 −0.03 (−3.48–3.37) *n* = 2355	−0.00 ± 1.00 −0.01 (−3.47–3.53) *n* = 2345	0.69
*Diff* adult height (SDS)	−0.00 ± 0.82 −0.00 (−2.78–3.05) *n* = 2355	−0.00 ± 0.80 −0.01 (−3.07–3.01) *n* = 2345	0.93

b			
*Birth size*			
*Q*_*birth*_ (SDS)	6.07 ± 0.57 6.07 (3.65–8.31) *n* = 2355	6.50 ± 0.60 6.51 (3.64–8.47) *n* = 2345	<0.0001
*Q*_*birth*_ (cm)	−0.12 ± 1.06 −0.16 (−4.21–3.92) *n* = 2355	−0.08 ± 1.00 −0.10 (−3.34–3.38) *n* = 2345	0.21
*DiffQ*_*birth*_ (SDS)	−0.12 ± 1.02 −0.14 (−3.83–3.91) *n* = 2355	−0.08 ± 0.97 −0.09 (−3.62–3.54) *n* = 2345	0.24
*E*_*birth*_ (cm)	45.37 ± 2.14 45.51 (31.38–51.23) *n* = 2355	44.05 ± 1.95 44.17 (32.39–50.32) *n* = 2345	<0.0001
*E*_*birth*_ (SDS)	−0.25 ± 1.12 −0.21 (−4.05–3.28) *n* = 2355	−0.25 ± 1.10 −0.25 (−4.64–3.69) *n* = 2345	0.73
*DiffE*_*birth*_ (SDS)	−0.25 ± 1.25 −0.24 (−5.02–3.77) *n* = 2355	−0.25 ± 1.26 −0.26 (−4.59–3.68) *n* = 2345	0.89
*QE*_*birth*_ (cm)	51.44 ± 2.40 51.59 (35.03–57.73) *n* = 2355	50.55 ± 2.18 50.67 (36.04–57.23) *n* = 2345	<0.0001
*QE*_*birth*_ (SDS)	−0.28 ± 1.14 −0.24 (−4.48–3.16) *n* = 2355	−0.26 ± 1.07 −0.25 (−4.75–3.65) *n* = 2345	0.98
*DiffQE*_*birth*_ (SDS)	−0.28 ± 1.19 −0.28 (−5.45–3.42) *n* = 2355	−0.26 ± 1.15 −0.26 (−4.16–3.42) *n* = 2345	0.88
*Early life (fetal-infancy) growth*			
*Q*_*max*_ (cm)	104.67 ± 8.06 104.34 (73.65–135.32) *n* = 2355	98.01 ± 7.79 97.87 (72.52–125.11) *n* = 2345	<0.0001
*Q*_*max*_ (SDS)	−0.12 ± 1.06 −0.16 (−4.21–3.92) *n* = 2355	−0.08 ± 1.00 −0.10 (−3.34–3.38) *n* = 2345	0.21
*DiffQ*_*max*_ (SDS)	−0.12 ± 1.02 −0.14 (−3.83–3.91) *n* = 2355	−0.08 ± 0.97 −0.09 (−3.62–3.54) *n* = 2345	0.25
*E*_*max*_ (cm)	65.12 ± 2.74 65.11 (56.03–74.82) *n* = 2355	62.91 ± 2.87 62.85 (54.65–73.30) *n* = 2345	<0.0001
*E*_*max*_ (SDS)	0.02 ± 1.02 0.02 (−3.35–3.62) *n* = 2355	0.03 ± 1.01 0.01 (−2.88–3.70) *n* = 2345	0.87
*DiffE*_*max*_ (SDS)	0.02 ± 1.17 0.05 (−3.99–4.11) *n* = 2355	0.03 ± 1.16 0.00 (−3.83–3.75) *n* = 2345	0.70
*QE*_*max*_ (cm)	169.79 ± 7.99 169.57 (135.77–198.52) *n* = 2355	160.92 ± 7.53 160.98 (135.10–189.84) *n* = 2345	<0.0001
*QE*_*max*_ (SDS)	−0.11 ± 1.06 −0.14 (−4.62–3.69) *n* = 2355	−0.07 ± 0.99 −0.07 (−3.47–3.74) *n* = 2345	0.18
*DiffQE*_*max*_ (SDS)	−0.11 ± 0.97 −0.12 (−3.58–3.39) *n* = 2355	−0.07 ± 0.89 −0.09 (−3.22–3.27) *n* = 2345	0.18
*E*_*timescale*_	1.00 ± 0.09 1.00 (0.69–1.35) *n* = 2355	1.01 ± 0.10 1.01 (0.69–1.37) *n* = 2345	0.0002
*Q*_*40w*_ (cm)	6.05 ± 0.47 6.03 (4.26–7.83) *n* = 2355	6.46 ± 0.51 6.45 (4.78–8.24) *n* = 2345	<0.0001
*Q*_*40w*_ (SDS)	−0.12 ± 1.06 −0.16 (−4.21–3.92) *n* = 2355	−0.08 ± 1.00 −0.10 (−3.34–3.38) *n* = 2345	0.21
*DiffQ*_*40w*_ (SDS)	−0.12 ± 1.02 −0.14 (−3.83–3.91) *n* = 2355	−0.08 ± 0.97 −0.09 (−3.62–3.54) *n* = 2345	0.24
*E*_*40w*_ (cm)	45.34 ± 1.76 45.41 (39.41–50.86) *n* = 2355	43.92 ± 1.69 43.91 (37.16–50.03) *n* = 2345	<0.0001
*E*_*40w*_ (SDS)	−0.25 ± 1.12 −0.21 (−4.04–3.27) *n* = 2355	−0.26 ± 1.10 −0.26 (−4.64–3.72) *n* = 2345	0.65
*DiffE*_*40w*_ (SDS)	−0.25 ± 1.25 −0.24 (−5.01–3.75) *n* = 2355	−0.26 ± 1.26 −0.27 (−4.60–3.72) *n* = 2345	0.82
*QE*_*40w*_ (SDS)	51.40 ± 1.84 51.46 (44.57–56.94) *n* = 2355	50.37 ± 1.76 50.40 (43.05–56.84) *n* = 2345	<0.0001
*QE*_*40w*_ (SDS)	−0.28 ± 1.14 −0.24 (−4.51–3.15) *n* = 2355	−0.27 ± 1.07 −0.25 (−4.73–3.68) *n* = 2345	0.94
*DiffQE*_*40w*_ (SDS)	−0.28 ± 1.20 −0.28 (−5.48–3.38) *n* = 2355	−0.27 ± 1.15 −0.27 (−4.18–3.40) *n* = 2345	0.94
*Age*_*E99*_ (years)	1.86 ± 0.22 1.85 (1.09–2.75) *n* = 2355	1.89 ± 0.24 1.88 (1.08–2.79) *n* = 2345	0.0002
*Q*_*E99*_ (cm)	22.37 ± 2.26 22.36 (15.19–31.33) *n* = 2355	23.58 ± 2.43 23.47 (15.32–37.18) *n* = 2345	<0.0001
*Q*_*E99*_ (SDS)	−0.12 ± 1.06 −0.16 (−4.21–3.92) *n* = 2355	−0.08 ± 1.00 −0.10 (−3.34–3.38) *n* = 2345	0.21
*DiffQ*_*E99*_ (SDS)	−0.12 ± 1.02 −0.14 (−3.83–3.91) *n* = 2355	−0.08 ± 0.97 −0.09 (−3.62–3.54) *n* = 2345	0.24
*E*_*E99*_ (cm)	64.47 ± 2.71 64.46 (55.47–74.07) *n* = 2355	62.28 ± 2.84 62.22 (54.10–72.57) *n* = 2345	<0.0001
*E*_*E99*_ (SDS)	0.02 ± 1.02 0.02 (−3.47–3.71) *n* = 2355	0.03 ± 1.01 0.01 (−2.94–3.67) *n* = 2345	0.88
*DiffE*_*E99*_ (SDS)	0.02 ± 1.16 0.05 (−4.10–4.03) *n* = 2355	0.03 ± 1.16 0.00 (−3.88–3.72) *n* = 2345	0.70
*QE*_*E99*_ (cm)	86.84 ± 4.10 86.78 (72.72–102.52) *n* = 2355	85.86 ± 4.34 85.73 (69.91–105.26) *n* = 2345	<0.0001
*QE*_*E99*_ (SDS)	−0.06 ± 1.03 −0.09 (−3.50–3.67) *n* = 2355	−0.04 ± 0.99 −0.05 (−4.16–4.03) *n* = 2345	0.38
*DiffQE*_*E99*_ (SDS)	−0.06 ± 0.99 −0.03 (−3.12–3.52) *n* = 2355	−0.04 ± 0.94 −0.05 (−3.28–3.01) *n* = 2345	0.67
*Early fetal−infancy growth differences*			
*ChangeQ*_*birth-40w*_ (SDS)	−0.00 ± 0.00 −0.00 (−0.00–0.00) *n* = 2355	−0.00 ± 0.00 −0.00 (−0.00–0.00) *n* = 2345	<0.0001
*ChangeE*_*birth-40w*_ (SDS)	−0.00 ± 0.04 −0.00 (−0.38–0.34) *n* = 2355	−0.01 ± 0.04 −0.00 (−0.27–0.66) *n* = 2345	0.0011
*ChangeQES*_*birth-40w*_ (SDS)	−0.00 ± 0.04 −0.00 (−0.31–0.31) *n* = 2355	−0.00 ± 0.04 −0.00 (−0.30–0.57) *n* = 2345	0.0017
*ChangeQ*_*40w-E99*_ (SDS)	0.00 ± 0.00 0.00 (−0.00–0.00) *n* = 2355	0.00 ± 0.00 0.00 (−0.00–0.00) *n* = 2345	0.13
*ChangeE*_*40w-E99*_ (SDS)	0.28 ± 1.15 0.22 (−3.70–4.69) *n* = 2355	0.29 ± 1.15 0.26 (−3.63–4.51) *n* = 2345	0.62
*ChangeQE*_*40w-E99*_ (SDS)	0.22 ± 1.07 0.20 (−3.45–3.99) *n* = 2355	0.22 ± 1.04 0.20 (−3.55–5.15) *n* = 2345	0.81
*Childhood growth differences*			
*ChangeQ*_*E99-P5*_ (SDS)	−0.00 ± 0.00 0.00 (−0.00–0.00) *n* = 2355	−0.00 ± 0.00 0.00 (−0.00–0.00) *n* = 2345	<0.0001
*ChangeE*_*E99-P5*_ (SDS)	65.10 ± 1.73 65.08 (59.50–71.11) *n* = 2355	62.88 ± 1.86 62.84 (57.59–69.64) *n* = 2345	<0.0001
*ChangeQE*_*E99-P5*_ (SDS)	−0.05 ± 0.70 −0.07 (−2.22–2.94) *n* = 2355	−0.03 ± 0.64 −0.04 (−2.25–2.20) *n* = 2345	0.26
*Childhood duration*			
*Age*_*E99-P5*_ (years)	9.96 ± 1.07 9.93 (6.53–13.98) *n* = 2355	7.97 ± 1.06 7.93 (4.48–13.01) *n* = 2345	<0.0001
*Pubertal growth*			
*P*_*max*_ (cm)	17.33 ± 3.88 17.51 (1.02–32.61) *n* = 2355	12.91 ± 3.73 12.87 (0.00–26.85) *n* = 2345	<0.0001
*P*_*max*_ (SDS)	0.02 ± 1.05 0.07 (−4.38–4.14) *n* = 2355	−0.01 ± 1.05 −0.03 (−3.70–3.92) *n* = 2344	0.15
*Timing and duration of puberty*			
*P*_*timescale*_	1.01 ± 0.05 1.00 (0.72–1.24) *n* = 2355	1.01 ± 0.04 1.01 (0.77–1.17) *n* = 2345	0.07
*Age*_*P5*_ (years)	11.82 ± 1.04 11.80 (8.57–15.86) *n* = 2355	9.86 ± 1.02 9.84 (6.61–14.82) *n* = 2345	<0.0001
*Age*_*P95*_ (years)	16.13 ± 1.04 16.09 (13.14–20.23) *n* = 2355	14.66 ± 1.01 14.64 (11.56–19.57) *n* = 2345	<0.0001
*Age*_*P5-P95*_ (years)	4.32 ± 0.21 4.30 (3.09–5.31) *n* = 2355	4.80 ± 0.19 4.78 (3.65–5.57) *n* = 2345	<0.0001
*Age*_*P50*_ (years)	13.84 ± 1.03 13.82 (10.70–17.90) *n* = 2355	12.09 ± 1.01 12.07 (8.91–17.03) *n* = 2345	<0.0001
*Age*_*TPHV*_ (years)	13.70 ± 1.04 13.67 (10.55–17.75) *n* = 2354	11.83 ± 1.01 11.81 (8.70–16.80) *n* = 2325	<0.0001
*Age*_*TPHV*_ (SDS)	−0.00 ± 1.00 −0.03 (−3.04–3.91) *n* = 2354	0.00 ± 1.00 −0.02 (−3.11–4.93) *n* = 2325	0.69
*Puberty growth differences*			
*ChangeP*_*P5-P95*_ (SDS)	0.01 ± 0.07 0.00 (−0.21–1.36) *n* = 2355	0.00 ± 0.04 −0.00 (−0.14–0.69) *n* = 2344	0.034
*ChangeT*_*P5-P95*_ (SDS)	−0.00 ± 0.58 −0.00 (−2.14–2.39) *n* = 2355	−0.03 ± 0.56 −0.03 (−2.49–1.94) *n* = 2344	0.13
*ChangeQES*_*P5-P95*_ (SDS)	−0.01 ± 0.33 −0.01 (−1.10–1.13) *n* = 2355	−0.02 ± 0.36 −0.04 (−1.40–1.44) *n* = 2344	0.13

Data are presented as mean ± standard deviation, median (range) and number of observations.

For test between two groups with respect to continuous variables Mann–Whitney U-test.

*SDS* standard deviation scores, *cm* centimeters, *Diff* the calculated differences between the individual’s length/height in SDS at the given timepoint and the individual mid-parental height in SDS_,_ i.e. the intrafamilial height difference, *Max* the maximal amplitude of the actual QEPS-function in centimeters and SDSs, or the timepoint when the function reaches its maximal amplitude, in years, *Change* the calculated growth difference in SDS of the actual QEPS-function between two different timepoints, *CI* confidence interval, *cm* centimeters, *SDS* standard deviation scores, *Diff adult height* the calculated difference between the individual’s adult height in SDS and the individual mid-parental height in SDS_,_ i.e. the intrafamilial height difference.

### Pubertal growth, *P*_*max*_*SDS*

#### Univariate explanatory analyses

**Fig. 2:**
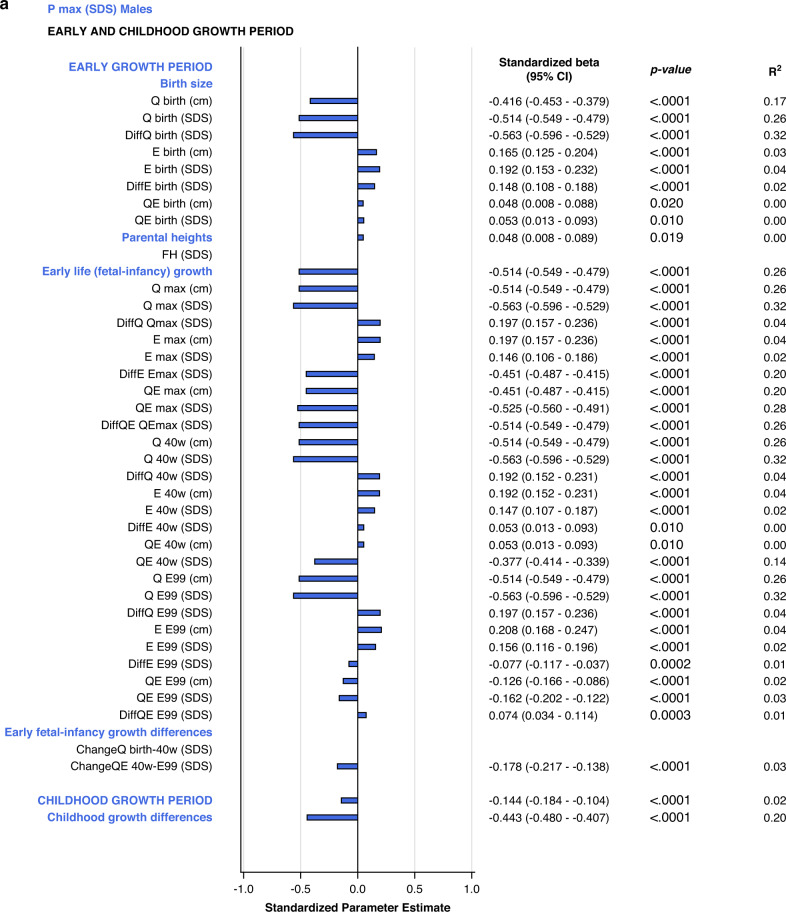

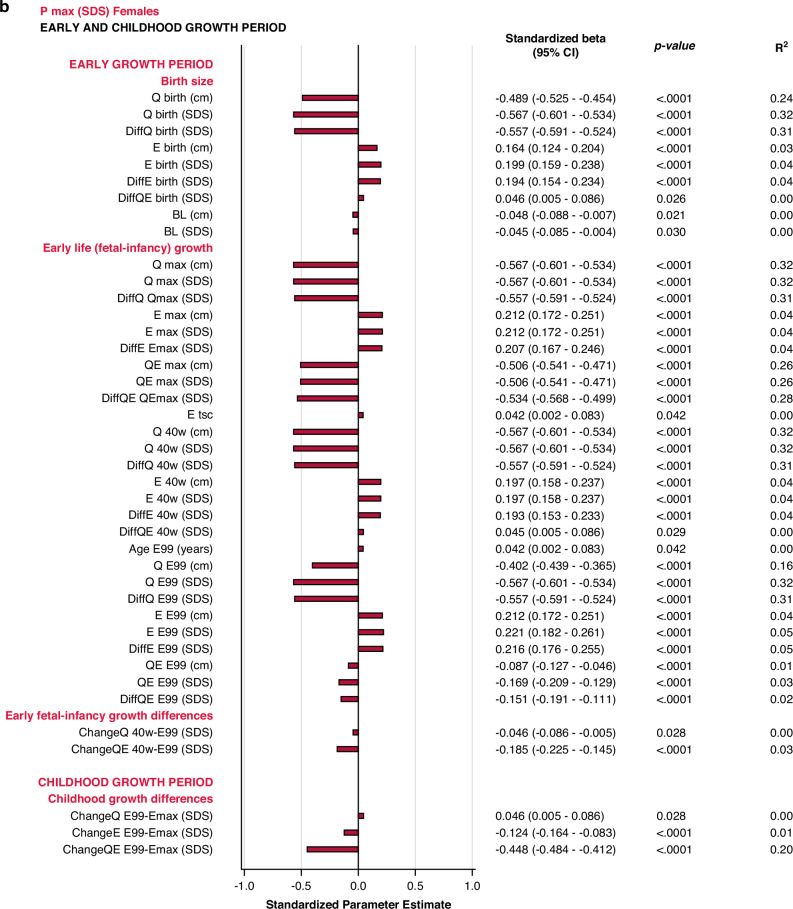
Bar graphs presenting results from the univariate linear regression models for *P*_*max*_*SDS* explained by selected variables separately for males (blue) and for females (red). All significant (*p* < 0.05) variables are presented with standardized beta (95% CI), *p*-value and R^2^. The bars represent the standardized beta (95% CI).

The specific early growth variables, described by the *E-*function, were strongly and significantly correlated with the specific, pubertal growth (*P*_*max*_*SDS*). The most influential variables were derived from the *Q-*function, representing basic growth. Correlations were significant for most *Q-*function variables, even the *QE*-function. The intrafamilial height difference, the *DiffSDSs* for *Q* and *QE* also correlated with the specific pubertal growth *(P*_*max*_*SDS)*. Parental heights or gestational age at birth could not explain the variations in *P*_*max*_*SDS*. Concordant results were obtained for males and females. There were no statistically significant interactions with sex following adjustment for Type I error. The models are presented in Fig. 2.

#### Multivariate models applied on different domains of explanatory variables

In the *birth size domain*, *Q*_*birth*_ and *E*_*birth*_ explained 28% and 33% of the variability in specific, pubertal growth (*P*_*max*_*SDS)* for males and females, respectively. *Q*_*birth*_ was negatively related to *P*_*max*_*SDS* whereas *E*_*birth*_ was positively related to *P*_*max*_*SDS*. The *early life growth domain* explained 37% of the specific, pubertal growth (*P*_*max*_*SDS)* for males, driven by *DiffQ*_*max*_*SDS*, standardized beta −0.53 (95% CI −0.57, −0.49), *p* < 0.0001, partial R^2^ 0.32. For females, 40% of the total variation in *P*_*max*_*SDS* could be explained by the *early life growth domain*, predominantly by *Q*, as *Q*_*max*_*SDS*, standardized beta -0.37 (95% CI −0.42, −0.33), *p* < 0.0001, partial R^2^ 0.32.

**Fig. 3:**
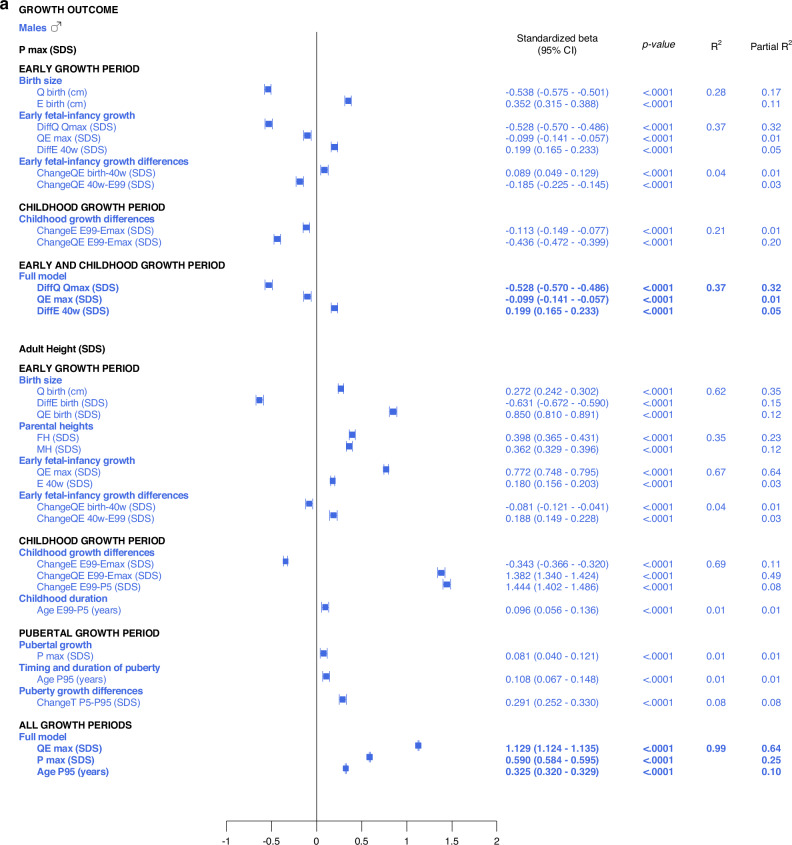

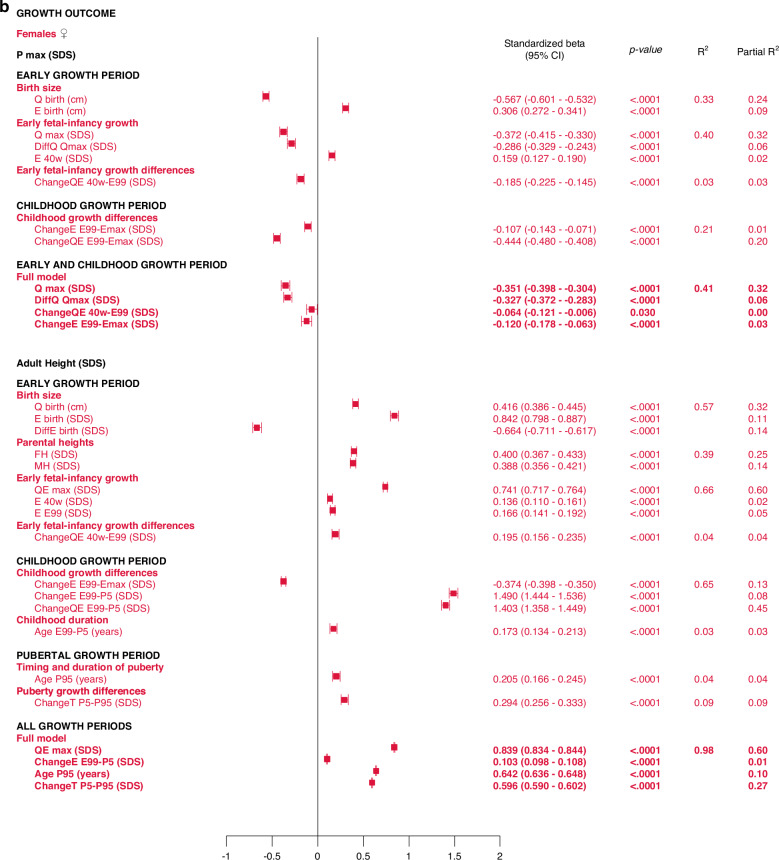
Forest plots presenting results from the multivariate linear regression models for *P*_*max*_*SDS* and *AH SDS*, separately for males (blue) and for females (red). All significant (*p* < 0.05) variables are presented with standardized beta (95% CI), *p*-value and R^2^. The Forest plots represent the standardized Beta (95% CI). The multivariate models per growth period are presented at the top and the multivariate total models at the bottom, in bold.

The *early life growth differences domain* contributed least to the explanation of *P*_*max*_*SDS*, 4% for males and 3% for females. Considering the *childhood growth differences domain*, changes in *E SDS* and *QE SDS* contributed equally to models for males and females, explaining 21% of the variation in *P*_*max*_*SDS*. These models are depicted in the forest plot, Fig. 3 and in Supplementary Table 2a.

#### Total, multivariate models including all domains

Taking all variables into account, the ones contributing independently and the most to the explanation of variability in specific pubertal growth (*P*_*max*_*SDS)* were those belonging to the *early life growth domain*, explaining 37% of the variation in males and 38% in females. Figure 3 and Supplementary Table 3a.

### Timing of pubertal growth, *Age*_*P5*_ and *Age*_*TPHV*_

#### Univariate explanatory analyses

There were associations, with a maximal R2 of 8%, between the pubertal timing outcomes, *Age*_*P5*_ (pubertal onset) and *Age*_*TPHV*_ (mid-puberty), and the explanatory variables studied. The highest R2s in the range of 7–8% were observed for various *DiffSDS* for *Q* and *QE* variables (intrafamilial height differences). In addition, there were correlations, with R2s of 1–2%, between *Age*_*P5*_ and variables from the birth size and parental heights domains, and likewise between *Age*_*TPHV*_ and variables from the birth size and parental heights domains. Gestational age could not explain variations in *Age*_*P5*_ and *Age*_*TPHV*_. There were no significant interactions with sex. The models are presented in Supplementary Figs. 2–5.

#### Multivariate models applied on different domains of explanatory variables

**Fig. 4:**
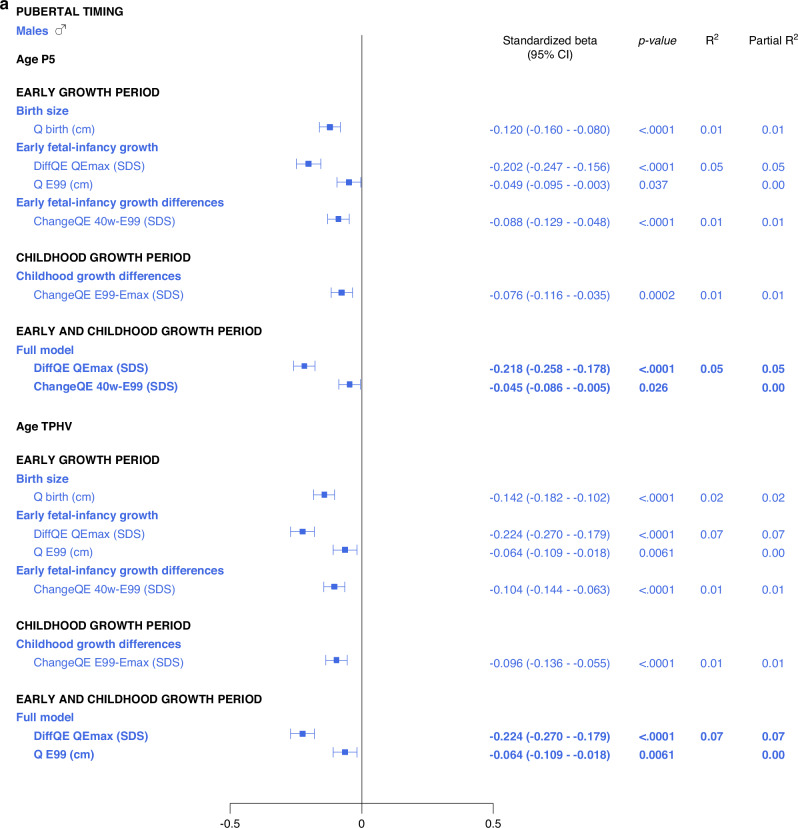

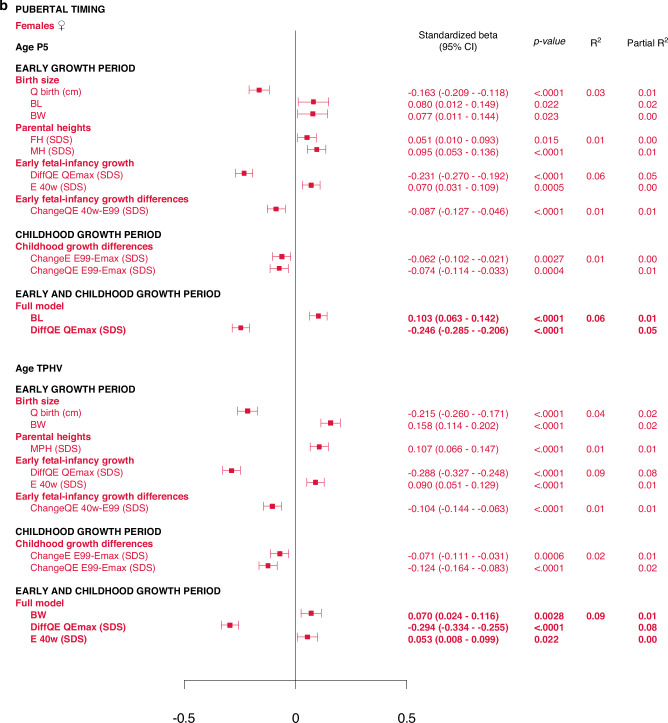
Forest plots presenting results from the multivariate linear regression models for *Age*_*P5*_ and *Age*_*TPHV*_, separately for males (blue) and for females (red). All significant (*p* < 0.05) variables are presented with standardized beta (95% CI), *p*-value and R^2^. The Forest plots represent the standardized beta (95% CI). The multivariate models per growth period are presented at the top and the multivariate total models at the bottom, in bold.

The results are presented as forest plots in Fig. 4, and Supplementary Tables 2b and 2c.

#### Total multivariate models including all domains

5% and 6% of the variance in *Age*_*P5*_, could be explained for males and females, respectively (Fig. 4, Supplementary Table 3b). Concerning *Age*_*TPHV*_, 7% and 9% of the variance could be explained for males and females, respectively (Fig. 4 and Supplementary Table 3c).

### Adult height, *AH SDS*

#### Univariate explanatory analyses

The most influential variables on adult height (*AH SDS*) were derived from the *Q-*function, representing basic growth. Even the specific early growth variables, described by the *E-*function were significantly associated with *AH SDS*. The *QE* and *Q*-functions alone explained between 48% and 64% of the variability in *AH SDS* for males, and between 41% and 60% of the variability in *AH SDS* for females. *Q*_*birth*_ explained 35% and 32%, whereas the *MPH SDS* explained 35% and 39% of the variability in *AH SDS* for males and females, respectively.

**Fig. 5:**
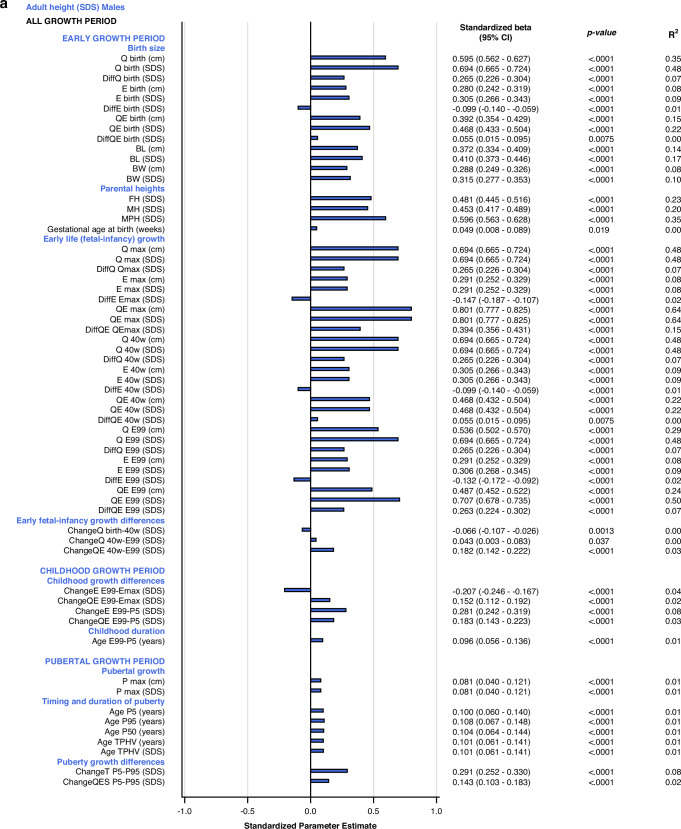

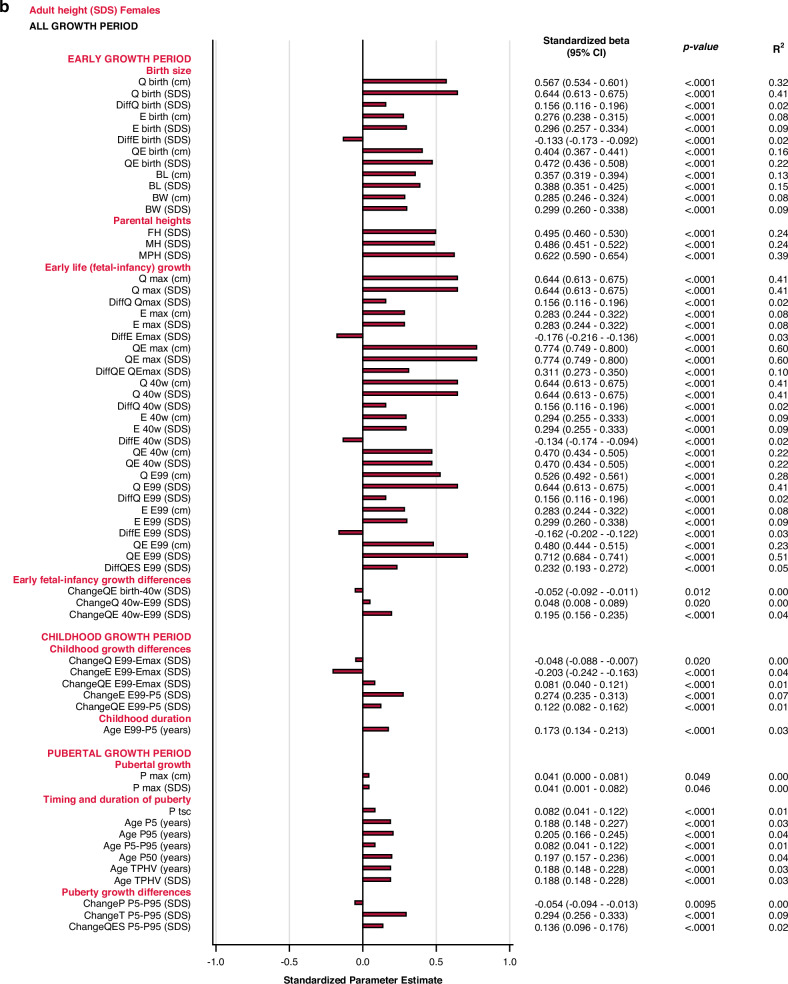
Bar graphs presenting results from the univariate linear regression models for *AH SDS* explained by selected variables separately for males (blue) and for females (red). All significant (*p* < 0.05) variables are presented with standardized beta (95% CI), *p*-value and R^2^. The bars represent the standardized beta (95% CI).

*Growth differences in early life and childhood* could explain 3–8% of the variation in adult height (*AH SDS*). The duration of childhood and the timing of puberty each explained 3%, of the variation in *AH SDS* for females, and 1% of the variation for males. The specific pubertal growth, *P*_*max*_*SDS*, explained the variability in *AH SDS to 0–1%*, whereas the change in total SDS during puberty, *Change T*_*P5-P99*_, explained 8% of the variability for males and 9% for females. Gestational age did not explain variations in *AH SDS*. The models are presented in Fig. 5.

#### Multivariate models applied on different domains of explanatory variables

The *birth size domain* explained 62% and 57%, whereas the *early life growth domain* explained 67% and 66% of the variability in adult height (*AH SDS)* for males and females, respectively. The *parental heights domain* explained 35–39% of the variability, dominated by the father’s heights in SDS that explained 23–25% of the variation in *AH SDS*. *Early life growth differences* explained 4% of the variability for both sexes, whereas *childhood growth differences* explained 69% of the variability for males and 65% for females. *Childhood duration* explained 1% and 3% of the variability in *AH SDS* for males and females, respectively. The specific pubertal growth, *P*_*max*_*SDS*, explained 1% for males and 0% for females. Age at the end of puberty, *Age*_*P95*_, explained 1% for males and 4% for females. The change in total SDS during puberty, *Change T*_*P5-P99*_, explained 8% and 9% of the variation in *AH SDS* for males and females, respectively. These models are depicted in the forest plot, Fig. 3 lower part, Supplementary Table 2d.

#### Total multivariate models including all domains (R2 expected to be close to 100%)

With the variables available retrospectively at adult height, 99% of the variability in *AH SDS* for males could be explained with three variables that all had a positive association with *AH SDS*: *QE*_*max*_*SDS* with partial R^2^ 64%, *P*_*max*_*SDS* with partial R^2^ 25% and *Age*_*P95*_ with partial R^2^ 10%. For females, 98% of the variability in *AH SDS* could be explained by four variables that were positively associated with *AH SDS*: *QE*_*max*_*SDS*, with partial R^2^ 60%, change in total SDS during puberty, *Change T*_*P5-P99*_, with partial R^2^ 27%, age at the end of puberty, *Age*_*P95*_, with partial R^2^ 10%, and change in *E* during childhood, *E*_*E99-P5*_*SDS*, with partial R^2^ 1%. The models are presented in Fig. 3 and Supplementary Table 3d.

## Discussion

### Principal findings

The aim of the study was to explore how growth during early life relates to pubertal growth and timing, and how growth during different growth periods explains variation in adult height, using the *QEPS*-growth-model. We hypothesized that growth during early life is related to both the timing and the amplitude of pubertal growth, as well as to the adult height.

The actual study indicated early life growth and specific pubertal growth are strongly connected. In multivariate models, birth size and early life growth explained 28–33% and 37–38%, respectively, of the variance in specific pubertal growth (*P*_*max*_*SDS)*. Our models could only explain the variation in pubertal timing to a small degree (6–9%). The findings obtained for *Age*_*P5*_ (pubertal onset) were in accordance with the *Age*_*TPHV*_ (mid-puberty) findings. The variability in adult height (*AH SDS*) could be explained by birth size (57–62%), early life growth (66–67%), and childhood growth (65–69%), and to a lesser degree by parental heights (35–39%). The change in height SDS during puberty explained 8–9% of variations in adult height for both sexes.

### Early life growth and timing of pubertal growth

Our models could only explain the variation in timing of pubertal growth to a small degree, and the influence of early growth on the timing of pubertal growth appears to be limited. In the literature, there are though, two well-described growth patterns^46,48^ that link early life growth to pubertal timing; *constitutional delay of growth and puberty*^46^ and *constitutional acceleration of growth*.^48^ Contrary to models used in these studies, the *QEPS*-model^41^ separates individual specific pubertal growth from ongoing, individual basic growth. The conflicting results might thus be due to these methodological differences. Early puberty is of clinical interest as it can lead to short stature due to premature closure of growth plates in long bones, mediated by estradiol,^4^ as well as to psychosocial difficulties,^47^ cardiometabolic diseases,^34^ and raised risk of certain cancers.^47^

Several studies have shown that individuals born SGA, especially females, are at risk of having an earlier pubertal onset.^20,21^ The currents study did not find any association between birth size and the timing of pubertal growth. However, this may be due to the characteristics of the individuals in our population. The cohort we studied was a healthy population in which all females had birthweights exceeding 1000 g. Pubertal timing can also be assessed in different ways. This study focused on the timing of pubertal growth, while some other studies have used pubertal milestones like menarche, gonadarche, thelarche, and pubarche.^17,43^ Most importantly, we have studied birth length and height gains in relation to pubertal timing, whereas most other studies focus on birth weight and weight gain. It is well known that rapid gains in weight in early life correlate with an earlier pubertal onset,^17–20^ but the evidence for a linkage between height gain and early pubertal timing remains sparce.^22,23^

Chronic stress in early life is believed not only to impair growth but also to predispose towards an earlier onset of puberty,^27,28^ likely through premature activation of neuroendocrine axes in early infancy.^27^ We hypothesized that preterm birth could be used as a marker for early life stress but found no associations between pubertal timing and gestational age. Mericq et al.^29^ found increased hormonal activity during the first months of life among infants born preterm, potentially predisposing them to altered pubertal trajectories.^29^ Meanwhile, the published evidence does not suggest that being born preterm leads to a significant acceleration in the onset of puberty,^31,32^ which is in accordance with our findings.

### Early life growth, pubertal growth and adult height

This study showed that early life growth is significantly associated with pubertal growth, as well as with adult height. In the total multivariate models, 28–33% of the variance in pubertal growth could be explained by birth size and 37–38% by early life growth. Surprisingly, specific pubertal growth, *P*_*max*_*SDS*_,_ had minimal influence on adult height. Additionally, the timing of the different growth periods, including puberty, was of minor importance for the variance of adult height. Our findings are in accordance with Prader’s^7^ findings in the 1990s, revealing that growth and maturation are two different, largely independent processes and that adult height depends mainly on prepubertal growth and that the timing of puberty has very little impact.^7^ Limony et al.^49^ showed that age and height at onset of the pubertal growth spurt affect the final height of children. However, they found a very low correlation (*r* = *0.04* and *r* = *0.05*) between the age at start of the pubertal growth spurt and final height,^49^ in our view indicating that the prepubertal height explains most of the variation in final height.

Even though growth in early life is strongly nutrition-dependent, the fact that maternal height is a major determinant of fetal size,^8^ highlights that there is an important genetic component in early life growth regulation. Both pubertal growth and adult height are strictly genetically regulated,^34–36^ and birth length is strongly associated with adult height.^37^ These findings, in line with ours, reveal that early life growth, pubertal growth and adult height probably share heritability, at least partially. It is estimated that 75–80% of adult stature is determined genetically.^36^ Recent genome-wide association studies have found over 400 height-associated loci, with cartilage being the most strongly implicated tissue system.^36^

While the genetic role in determining adult height is unquestionable, adult height is still an accurate indicator of early life exposure to factors such as nutrition.^39^ Individuals who experienced famine throughout their prenatal life and early childhood exhibited a notable reduction in adult height.^50^ It has additionally been shown that growth failure prior to 12 months of age impairs final stature.^40^ From a clinical perspective, even children with diseases that predispose them to malnutrition, like cystic fibrosis, short bowel syndrome or anorexia, are at risk of being short as adults.^39^ Undernutrition induces a state of growth hormone resistance, increases cortisol levels, and decreases levels of thyroid hormone as well as androgen and estrogen, which may all contribute to the slowing of growth.^50^ Importantly, poor growth among preterm born children might also be due to growth hormone resistance.^4^ It is well known that severe prematurity can lead to a reduction in adult height.^37^ In our study, we did not find any association between gestational age and adult height. This is probably due to a low number of children with very (*n* = 16) and extremely (*n* = 1) preterm birth in our material. Our findings are thus more in line with studies of children born moderately preterm (32 + 0 to 34 + 0 weeks/days of gestation), showing an unaffected adult height.^51,52^

### Exploring early growth, pubertal growth and timing with the QEPS-model

The *QEPS*-model^41^ and the ICP (Infancy–Childhood–Puberty) -model^6^ are the only two growth models that enable studies of early life growth. The frequently used SITAR (Super-Imposition by Translation And Rotation) -model^53^ can only describe growth from around 5 years of age, and thus not early life growth. In contrast to the ICP model, the *QEPS*-model provides flexibility in the tempo and amplitude of early life growth (*E*-function) and pubertal growth (*P*-function). Moreover, the *QEPS*-model enables detailed study of specific early life growth (*E*-function) and specific pubertal growth (*P*-function), separated from the basic ongoing *Q*-function growth.^41^ However, the *QEPS*-model describes the early life growth period as a continuum and does not naturally differentiate fetal growth and infancy growth.

Traditionally, the timing of pubertal growth is assessed as age at PHV,^44,45^ this age falls in the middle of puberty, approximately 24 months after its onset.^44^ Age at PHV corresponds to our variable *Age*_*TPHV*_. We additionally introduced the concept of *Age*_*P5*_, the age when 5% of specific pubertal growth is achieved, as a marker of the onset of pubertal growth. For most individuals, 5% of specific, pubertal growth corresponds to a 6–9 mm increase in height.^44^ *Age*_*P5*_ is an absolute defined starting point, with reliability and a low CI,^44^ chosen because of its clinical usefulness. However, as *Age*_*TPHV*_ and *Age*_*P5*_ are strongly associated,^44^ our results for these two outcomes are mainly concordant.

Genetic insights by Bradfield et al.^35^ have elucidated that independent loci signals can influence either specific pubertal growth, or basic growth from birth to adulthood.^35^ This finding support our idea of specific pubertal growth, *P*_*max*_, that can be separated from basic, ongoing growth. By using the *QEPS*-model^41^ to divide total pubertal growth (*T*_*P5-P95*_*)* into basic growth during puberty (*QES*_*P5-P95*_) and specific pubertal growth (*P*_*P5-P95*_), pubertal growth can be studied in a fundamental new way.

### Strengths and limitations

This study has many strengths, primary, the adult height of all participants was measured by the same research team.^26^ Furthermore, the study group was population-based, representing the broad socioeconomic diversity present in the Gothenburg area. The study included children with optimal, healthy growth, sourced from the GrowUp_1974&1990_Gothenburg cohorts,^25,26,42^ from which individuals were used to create national Swedish growth references.^25,42^ We analyzed dropouts among 993 children meeting inclusion criteria but lacking parental heights; they statistically matched the study group (Supplementary Table 1). Consequently, our results should be highly generalizable.

In this study, we have attempted to fit the dynamic, biological process of human growth into a mathematical model which therefore has some limitations; our findings may not directly translate into clinical applications. The functions of the *QEPS*-model^41^ are derived from the total growth curve, obtained when an individual’s growth is finished. Consequently, the early life growth functions depend on the total, individual growth.^41^

The study population’s homogeneity means that extreme group representation in terms of birth size and gestational age is limited, thus restricting the possible subgroup analyses. However, including prematurely and post-term born children enhances generalizability, unlike previous growth studies of the GrowUp_1974&1990_Gothenburg cohorts.^25,26,42^ The study population consists of two different cohorts born 16 years apart. We know that there is an ongoing secular trend in the Swedish population,^43^ and this could potentially be adjusted for. However, we assessed variations between the individuals within the cohorts and found these to be much greater than the variations between the cohorts. The study participants were born in 1974 and 1990. This should be considered, especially when studying individuals born preterm or with a small birth size, given the immense improvements of neonatal care in the past decades.

### Future perspectives

It is widely acknowledged that compromised early growth, whether assessed in centimeters or grams, serves as a significant indicator of developmental perturbations, linked to future health risks.^1,3^ Additionally, Barker^2^ and many others,^5,17^ have shown that rapid weight gain during infancy, particularly following fetal undernourishment, is associated with an increased susceptibility to metabolic disorders in adulthood.^2,5,17^ However, the relationship between accelerated length growth in early life and future health risks lacks definitive evidence.^5^

It has been shown previously that both early life and pubertal growth correlate with future health outcomes.^5^ Interestingly, Bradfield et al.^35^ state that there might not be a single optimal pubertal growth pattern. They found one genetic pattern with diminished growth between 8–14 years of age and adult age that was highly correlated with risk for coronary heart disease. Meanwhile, they found another pattern with great pubertal growth, associated with cardiac dysrhythmias.^35^ Likewise, tall adult stature has been associated with better cognitive abilities and superior earning capacity, but also with higher cancer risk.^39,54^ One could speculate that there are distinct, genetically determined growth trajectories that are differentially associated with disease risks, and that reduced growth in relation to the individual’s growth potential signals injury and an additional risk for adverse health outcomes.

Future research could explore the link between early growth and long-term health outcomes, considering factors such as weight development alongside height. Expanding studies to include larger and more diverse populations with different ethnicities, and studying cohorts of preterm infants born today, could provide further insights into growth patterns and health outcomes. It would also be of great interest to perform studies of individualized prediction of adult height and future health, related to growth.

## Conclusion

Our findings indicate that early life growth is strongly associated with the variability in pubertal growth, and adult height, but not with the timing of pubertal growth. Overall, this study highlights the importance of early life growth as a marker for future growth, development, and health.

## Supplementary information

Supplemental Figure 1

Supplemental Figure 2

Supplemental Figure 3

Supplemental Figure 4

Supplemental Figure 5

Supplemental Table 1

Supplemental Table 2a

Supplemental Table 2b

Supplemental Table 2c

Supplemental Table 2d

Supplemental Table 3a

Supplemental Table 3b

Supplemental Table 3c

Supplemental Table 3d

## Data Availability

The present dataset for the analyses was made as described in the method section. This data used for the present dataset was after administrative permissions obtained from the ‘GrowUp Gothenburg study Database’, and is now a part of this database, which is stored in a server at the Gothenburg University. Swedish Data protection Act (1998:204) does not permit sensitive data on humans (like the GrowUp Questionnaires) to be openly shared. However, the authors are positive to collaborate with researchers worldwide. The data are available upon request from anton.holmgren@regionhalland.se; depending on the research question, ethical approval might be required.
