# Supplementary material for: Early life growth is related to pubertal growth and adult height – a QEPS-model analysis
Source: Pediatr Res. 2025 Feb 25;98(4):1339–57. doi: 10.1038/s41390-025-03939-9 (PMC12549337; doi:10.1038/s41390-025-03939-9)

Supplemental Figure 1: Study flow chart for the study population from the Combo GrowUp Gothenburg 1974/1990 cohort

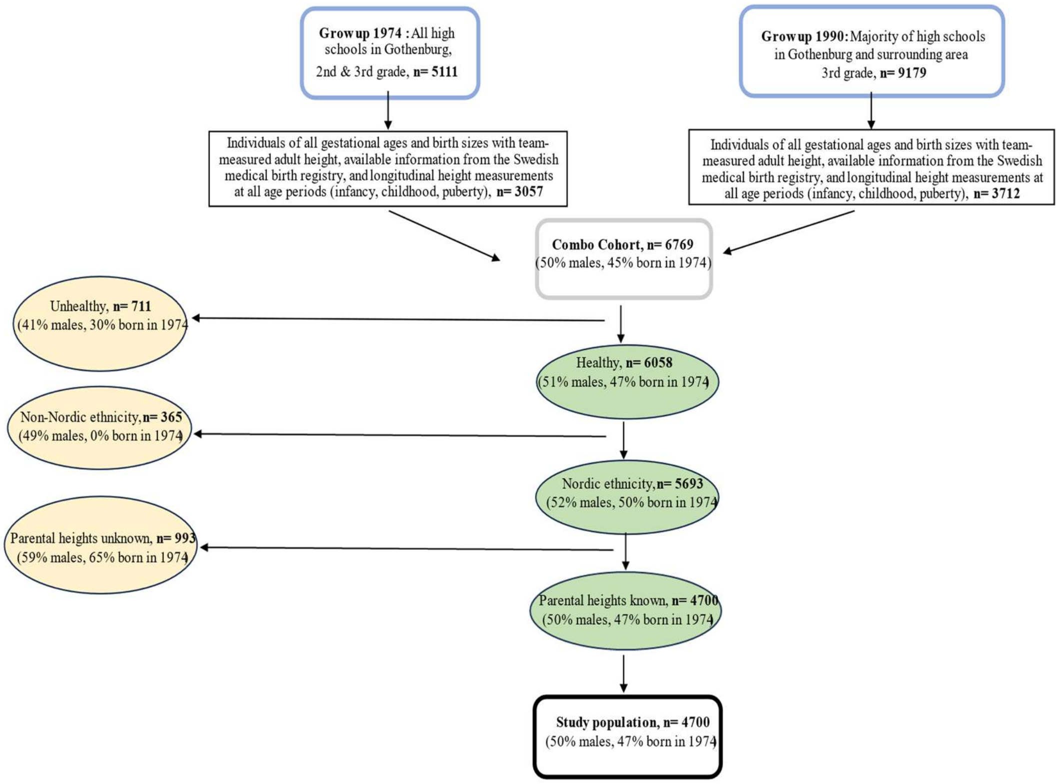

Supplement: Supplementary file 1 — Supplemental Figure 1 [file 41390_2025_3939_MOESM1_ESM.pdf]
