# Supplementary figures and images for: Early life growth is related to pubertal growth and adult height – a QEPS-model analysis
Source: Pediatr Res. 2025 Feb 25;98(4):1339–57. doi: 10.1038/s41390-025-03939-9 (PMC12549337; doi:10.1038/s41390-025-03939-9)

**Supplemental Figure 2: Univariable linear regression models for *AgeP5* for males**

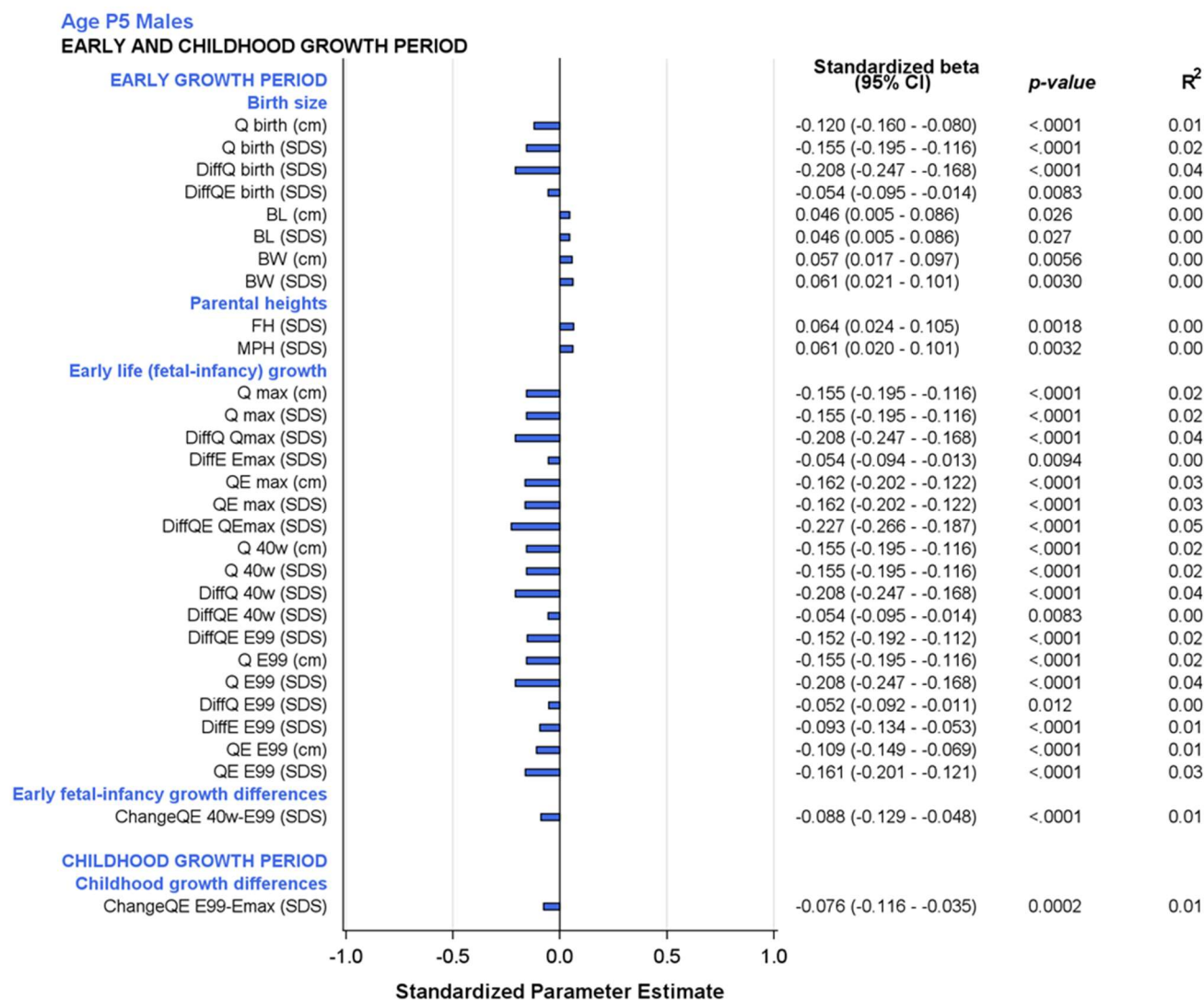

Supplement: Supplementary file 2 — Supplemental Figure 2 [file 41390_2025_3939_MOESM2_ESM.pdf]

**Supplemental Figure 3: Univariable linear regression models for *AgeP5* for females**

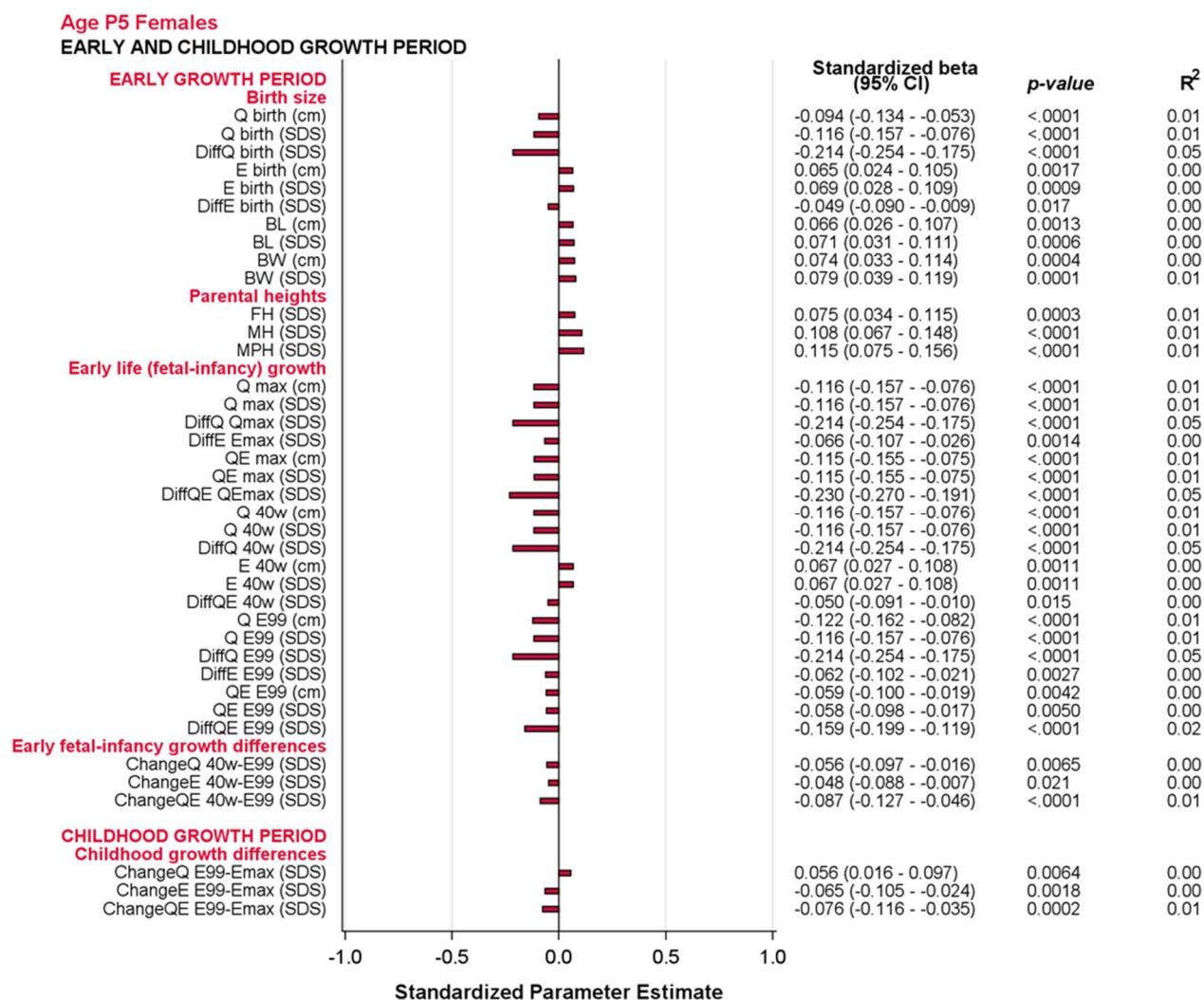

Supplement: Supplementary file 3 — Supplemental Figure 3 [file 41390_2025_3939_MOESM3_ESM.pdf]

**Supplemental Figure 4: Univariable linear regression models for *AgeTPHV* for males**

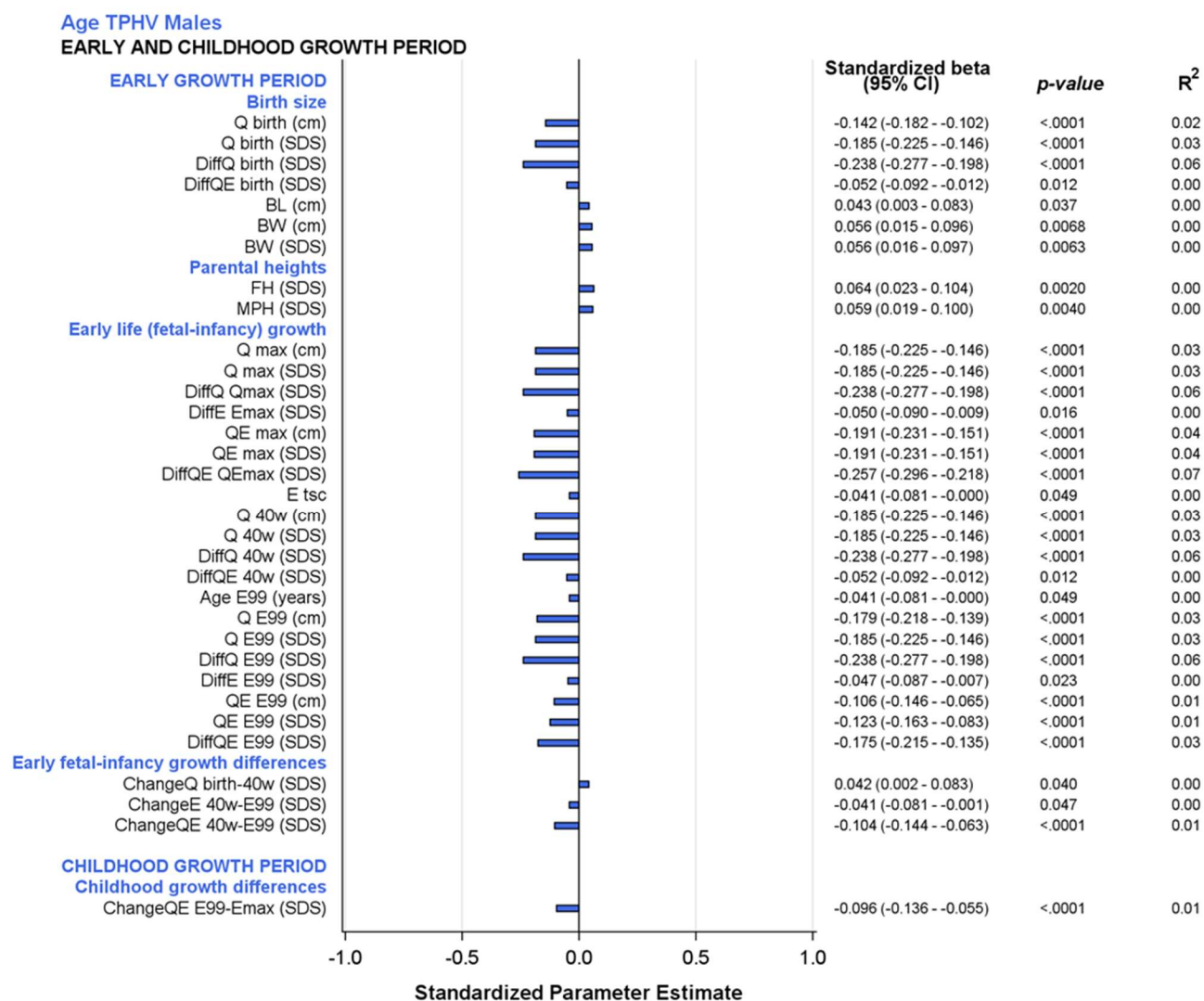

Supplement: Supplementary file 4 — Supplemental Figure 4 [file 41390_2025_3939_MOESM4_ESM.pdf]

**Supplemental Figure 5: Univariable linear regression models for *AgeTPHV* for females**

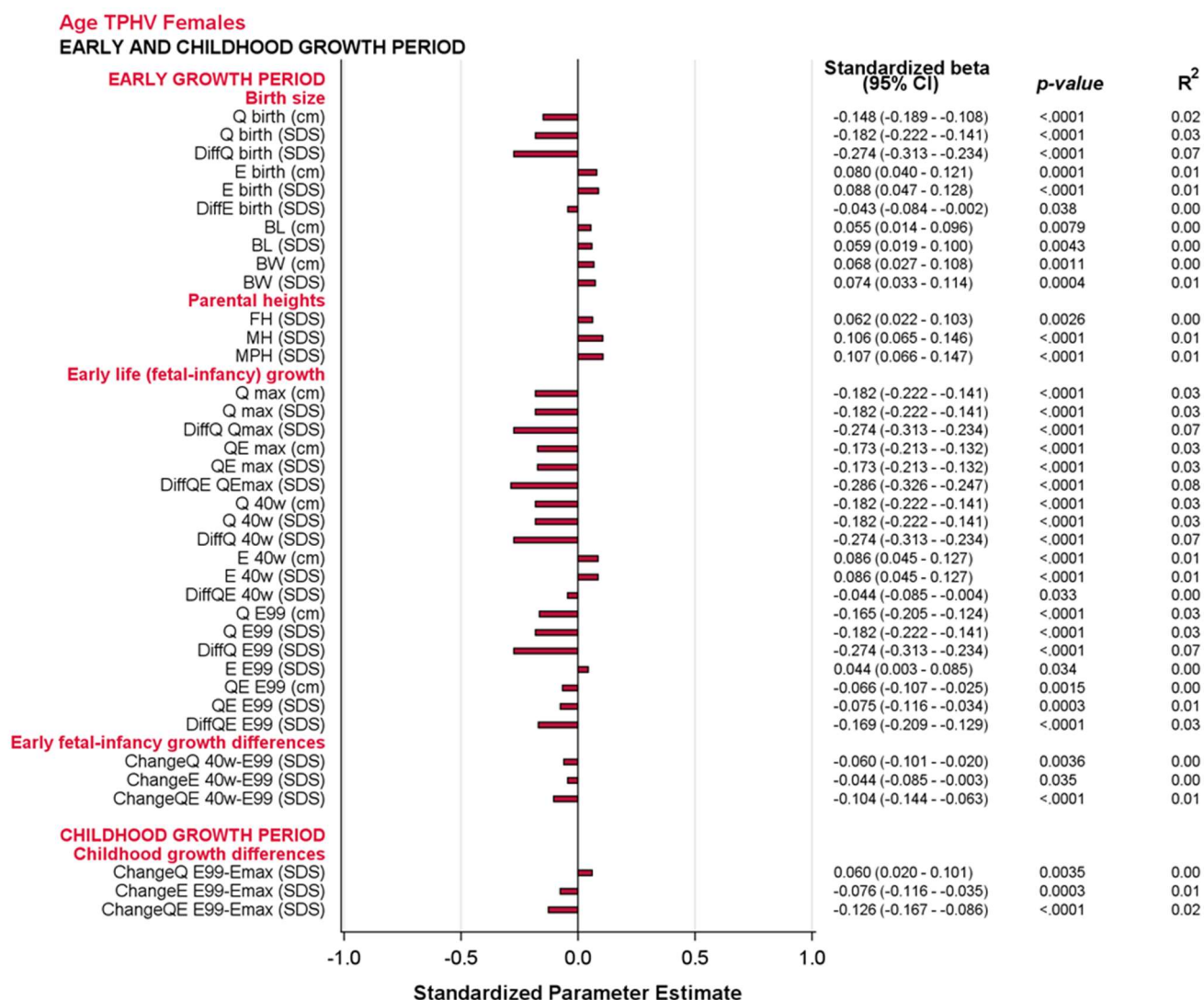

Supplement: Supplementary file 5 — Supplemental Figure 5 [file 41390_2025_3939_MOESM5_ESM.pdf]
