# Supplementary material for: Early life growth is related to pubertal growth and adult height – a QEPS-model analysis
Source: Pediatr Res. 2025 Feb 25;98(4):1339–57. doi: 10.1038/s41390-025-03939-9 (PMC12549337; doi:10.1038/s41390-025-03939-9)
Supplement: Supplementary file 7 — Supplemental Table 2a [file 41390_2025_3939_MOESM7_ESM.pdf]

Supplemental Table 2a: Multivariable linear regression models for ***P_{max}SDS*** (gain in adult height in standard deviation scores due to specific pubertal *P*-function growth) with explanatory variables clustered according to information available at each growth period.

Abbreviations: *SDS*, standard deviation scores; *cm*, centimeters

Diff, the calculated differences between the individual's length/height in SDS at the given timepoint and the individual mid-parental height in SDS, i.e. the intrafamilial height difference.

Max, the maximal amplitude of the actual QEPS-function in centimeters and SDSs, or the timepoint when the function reaches its maximal amplitude, in years.

Change, the calculated growth difference in SDS of the actual QEPS-function between two different timepoints.

		Male					Female				
**Domain**	**Variable**	**Standardized beta (95% CI)**	**p-value**	**R2**	**Partial R2**	**VIF**	**Standardized beta (95% CI)**	**p-value**	**R2**	**Partial R2**	**VIF**
Early life (fetal-infancy) growth	E_{birth} (SDS)	0.213 (0.179 - 0.246)	<.0001	0.31	0.05	1.00	0.193 (0.160 - 0.225)	<.0001	0.36	0.04	1.00
	Q_{max} (SDS)	-0.522 (-0.556 - -0.489)	<.0001		0.26	1.00	-0.565 (-0.598 - -0.533)	<.0001		0.32	1.00
Childhood	*Change* $E_{E99\text{-}Emax}$ (SDS)	-0.113 (-0.149 - -0.077)	<.0001	0.21	0.01	1.00	-0.107 (-0.143 - -0.071)	<.0001	0.21	0.01	1.00
	Change $QE_{E99\text{-}Emax}$ (SDS)	-0.436 (-0.472 - -0.399)	<.0001		0.20	1.00	-0.444 (-0.480 - -0.408)	<.0001		0.20	1.00
Parental heights and *Diff SDSs*	*Diff*E_{birth} (SDS)	0.223 (0.191 - 0.256)	<.0001	0.37	0.05	1.02	0.266 (0.234 - 0.299)	<.0001	0.38	0.07	1.02
	DiffQ_{max} (SDS)	-0.591 (-0.624 - -0.559)	<.0001		0.32	1.02	-0.590 (-0.622 - -0.558)	<.0001		0.31	1.02
Beta estimates are standardized both for the dependent and the independent variable.											
