# Supplementary material for: Early life growth is related to pubertal growth and adult height – a QEPS-model analysis
Source: Pediatr Res. 2025 Feb 25;98(4):1339–57. doi: 10.1038/s41390-025-03939-9 (PMC12549337; doi:10.1038/s41390-025-03939-9)
Supplement: Supplementary file 8 — Supplemental Table 2b [file 41390_2025_3939_MOESM8_ESM.pdf]

Supplemental Table 2b: Multivariable linear regression models for ***Age$_{P5}$*** (age at which 5% of the *P*-function growth is reached, in this study used as age at pubertal onset) with explanatory variables clustered according to information available at each growth period.

Abbreviations: *SDS*, standard deviation scores; *cm*, centimeters

Diff, the calculated differences between the individual's length/height in SDS at the given timepoint and the individual mid-parental height in SDS, i.e. the intrafamilial height difference.

Max, the maximal amplitude of the actual QEPS-function in centimeters and SDSs, or the timepoint when the function reaches its maximal amplitude, in years.

Change, the calculated growth difference in SDS of the actual QEPS-function between two different timepoints.

		Male				Female			
**Domain**	**Variable**	**Standardized beta (95% CI)**	**p-value**	**R2**	**VIF**	**Standardized beta (95% CI)**	**p-value**	**R2**	**VIF**
Birth size	Q_{birth} (cm)	-0.120 (-0.160 - -0.080)	<.0001	0.01	1.00	-0.163 (-0.209 - -0.118)	<.0001	0.03	1.29
	Birth length (cm)					0.080 (0.012 - 0.149)	0.022		2.95
	Birth weight (grams)					0.077 (0.011 - 0.144)	0.023		2.79
Parental heights and *DiffSDSs*	Father's height (cm)	0.062 (0.022 - 0.103)	0.0025	0.00	1.00	0.049 (0.008 - 0.091)	0.020	0.01	1.07
	Mother's height (cm)					0.093 (0.051 - 0.134)	<.0001		1.07
Early life (fetal-infancy) growth	Q_{max} (SDS)	-0.101 (-0.150 - -0.052)	<.0001	0.03	1.53	-0.070 (-0.118 - -0.021)	0.0047	0.02	1.46
	Q_{E99} (SDS)	-0.093 (-0.142 - -0.044)	0.0002		1.53	-0.083 (-0.131 - -0.034)	0.0008		1.46
Early life (fetal-infanc)y growth differences	*Change* $Q_{40w-E99}$ (SDS)	-0.134 (-0.174 - -0.094)	<.0001	0.02	1.00	-0.108 (-0.149 - -0.068)	<.0001	0.01	1.00
Childhood growth differences	*Change* Q_{E99-P5} (SDS)	0.940 (0.907 - 0.973)	<.0001	0.57	1.57	0.992 (0.965 - 1.019)	<.0001	0.69	1.44
	Change QE_{E99-P5} (SDS)	-0.651 (-0.684 - -0.618)	<.0001		1.57	-0.628 (-0.655 - -0.601)	<.0001		1.44
Beta estimates are standardized both for the dependent and the independent variable.									
