# Supplementary material for: Early life growth is related to pubertal growth and adult height – a QEPS-model analysis
Source: Pediatr Res. 2025 Feb 25;98(4):1339–57. doi: 10.1038/s41390-025-03939-9 (PMC12549337; doi:10.1038/s41390-025-03939-9)
Supplement: Supplementary file 9 — Supplemental Table 2c [file 41390_2025_3939_MOESM9_ESM.pdf]

Supplemental Table 2c: Multivariable linear regression models for ***Age_{TPHV}*** (age at peak height velocity of the total growth curve) with explanatory variables clustered according to information available at each growth period.

Abbreviations: *SDS*, standard deviation scores; *cm*, centimeters

Diff, the calculated differences between the individual's length/height in SDS at the given timepoint and the individual mid-parental height in SDS, i.e. the intrafamilial height difference.

Max, the maximal amplitude of the actual QEPS-function in centimeters and SDSs, or the timepoint when the function reaches its maximal amplitude, in years.

Change, the calculated growth difference in SDS of the actual QEPS-function between two different timepoints.

		Male				Female			
**Domain**	**Variable**	**Standardized beta (95% CI)**	**p-value**	**R2**	**VIF**	**Standardized beta (95% CI)**	**p-value**	**R2**	**VIF**
Birth size	Q_{birth} (cm)	-0.142 (-0.182 - -0.102)	<.0001	0.02	1.00	-0.215 (-0.260 - -0.171)	<.0001	0.04	1.22
	Birth weight (grams)					0.158 (0.114 - 0.202)	<.0001		1.22
Parental heights and *DiffSDSs*	Mother's height (cm)	0.028 (-0.012 - 0.069)	0.17	0.00	1.00	0.103 (0.062 - 0.143)	<.0001	0.01	1.00
Early life (fetal-infanc)y growth	Q_{max} (cm)	-0.123 (-0.172 - -0.074)	<.0001	0.04	1.53				
	Q_{40w} (cm)					-0.176 (-0.216 - -0.135)	<.0001	0.04	1.02
	E_{40w} (cm)					0.085 (0.045 - 0.125)	<.0001		1.00
	Q_{E99} (cm)	-0.107 (-0.156 - -0.058)	<.0001		1.53				
	QE_{E99} (cm)					-0.043 (-0.083 - -0.003)	0.036		1.02
Early life (fetal-infancy) growth differences	*Change* $Q_{40w\text{-}E99}$ (SDS)	-0.157 (-0.197 - -0.117)	<.0001	0.02	1.00	-0.140 (-0.181 - -0.100)	<.0001	0.02	1.00
Childhood growth differences	*Change* $Q_{E99\text{-}P5}$ (SDS)	0.527 (0.493 - 0.561)	<.0001	0.28	1.00	0.973 (0.945 - 1.001)	<.0001	0.67	1.42
	Change $QE_{E99\text{-}P5}$ (SDS)					-0.663 (-0.692 - -0.635)	<.0001		1.42
Beta estimates are standardized both for the dependent and the independent variable.									
