# Supplementary material for: Early life growth is related to pubertal growth and adult height – a QEPS-model analysis
Source: Pediatr Res. 2025 Feb 25;98(4):1339–57. doi: 10.1038/s41390-025-03939-9 (PMC12549337; doi:10.1038/s41390-025-03939-9)
Supplement: Supplementary file 10 — Supplemental Table 2d [file 41390_2025_3939_MOESM10_ESM.pdf]

Supplemental Table 2d: Multivariable linear regression models for ***Adult height SDS*** with explanatory variables clustered according to information available at each growth period.

Abbreviations: *SDS*, standard deviation scores; *cm*, centimeters

Diff, the calculated differences between the individual's length/height in SDS at the given timepoint and the individual mid-parental height in SDS, i.e. the intrafamilial height difference.

Max, the maximal amplitude of the actual QEPS-function in centimeters and SDSs, or the timepoint when the function reaches its maximal amplitude, in years.

Change, the calculated growth difference in SDS of the actual QEPS-function between two different timepoints.

		**Male**				**Female**			
**Domain**	**Variable**	**Standardized beta (95% CI)**	**p-value**	**R2**	**VIF**	**Standardized beta (95% CI)**	**p-value**	**R2**	**VIF**
Birth size	Q_{birth} (cm)	0.537 (0.507 - 0.567)	<.0001	0.45	1.03	0.518 (0.487 - 0.549)	<.0001	0.42	1.02
	Birth length (SDS)	0.329 (0.298 - 0.359)	<.0001		1.03	0.314 (0.283 - 0.345)	<.0001		1.02
Parental heights and *DiffSDSs*	Father's height (cm)	0.401 (0.368 - 0.435)	<.0001	0.36	1.06	0.399 (0.367 - 0.432)	<.0001	0.39	1.07
	Mother's height (cm)	0.361 (0.328 - 0.394)	<.0001		1.06	0.388 (0.355 - 0.420)	<.0001		1.07
Early life (fetal-infancy) growth	Q_{max} (cm)	0.787 (0.764 - 0.810)	<.0001	0.68	1.04	0.594 (0.571 - 0.618)	<.0001	0.67	1.02
	E_{max} (cm)	0.447 (0.423 - 0.470)	<.0001		1.04				
	E_{40w} (cm)					0.309 (0.286 - 0.333)	<.0001		1.00
	QE_{E99} (cm)					0.399 (0.375 - 0.422)	<.0001		1.02
Early life (fetal-infancy) growth differences	*Change* $Q_{40w\text{-}E99}$ (SDS)	0.851 (0.801 - 0.902)	<.0001	0.32	2.32	0.428 (0.391 - 0.464)	<.0001	0.18	1.00
	Change $E_{40w\text{-}E99}$ (SDS)	-0.555 (-0.606 - -0.504)	<.0001		2.32				
Childhood growth differences	*Change* $Q_{E99\text{-}P5}$ (SDS)	0.754 (0.726 - 0.781)	<.0001	0.59	1.12	0.735 (0.706 - 0.764)	<.0001	0.55	1.15
	Change $E_{E99\text{-}P5}$ (SDS)	0.542 (0.514 - 0.569)	<.0001		1.12	0.552 (0.522 - 0.581)	<.0001		1.15
Beta estimates are standardized both for the dependent and the independent variable.									
