# Supplementary material for: Early life growth is related to pubertal growth and adult height – a QEPS-model analysis
Source: Pediatr Res. 2025 Feb 25;98(4):1339–57. doi: 10.1038/s41390-025-03939-9 (PMC12549337; doi:10.1038/s41390-025-03939-9)
Supplement: Supplementary file 11 — Supplemental Table 3a [file 41390_2025_3939_MOESM11_ESM.pdf]

Supplemental Table 3a: Multivariable total models for ***P_{max}SDS*** (gain in adult height in standard deviation scores due to specific pubertal *P*-function growth).

Abbreviations: *SDS*, standard deviation scores; *cm*, centimeters

Diff, the calculated differences between the individual's length/height in SDS at the given timepoint and the individual mid-parental height in SDS, i.e. the intrafamilial height difference.

Max, the maximal amplitude of the actual QEPS-function in centimeters and SDSs, or the timepoint when the function reaches its maximal amplitude, in years.

Change, the calculated growth difference in SDS of the actual QEPS-function between two different timepoints.

	Male					Female				
**Variable**	**Standardized beta (95% CI)**	**p-value**	**R2**	**Partial R2**	**VIF**	**Standardized beta (95% CI)**	**p-value**	**R2**	**Partial R2**	**VIF**
Q_{max} (SDS)						-0.351 (-0.398 - -0.304)	<.0001	0.41	0.32	2.27
DiffQ_{max} (SDS)	-0.528 (-0.570 - -0.486)	<.0001	0.37	0.32	1.72	-0.327 (-0.372 - -0.283)	<.0001		0.06	2.04
QE_{max} (SDS)	-0.099 (-0.141 - -0.057)	<.0001		0.01	1.74					
DiffE_{40w} (SDS)	0.199 (0.165 - 0.233)	<.0001		0.05	1.12					
Change $QE_{40w\text{-}E99}$ (SDS)						-0.064 (-0.121 - -0.006)	0.030		0.00	3.42
Change $E_{E99\text{-}Emax}$ (SDS)						-0.120 (-0.178 - -0.063)	<.0001		0.03	3.34
Beta estimates are standardized both for the dependent and the independent variable.										
