# Supplementary material for: Early life growth is related to pubertal growth and adult height – a QEPS-model analysis
Source: Pediatr Res. 2025 Feb 25;98(4):1339–57. doi: 10.1038/s41390-025-03939-9 (PMC12549337; doi:10.1038/s41390-025-03939-9)
Supplement: Supplementary file 12 — Supplemental Table 3b [file 41390_2025_3939_MOESM12_ESM.pdf]

Supplemental Table 3b: Multivariable total models for $\boldsymbol{Age_{P5}}$ (age at which 5% of the *P*-function growth is reached, in this study used as age at pubertal onset).

Abbreviations: *SDS*, standard deviation scores; *cm*, centimeters

Diff, the calculated differences between the individual's length/height in SDS at the given timepoint and the individual mid-parental height in SDS, i.e. the intrafamilial height difference.

Max, the maximal amplitude of the actual QEPS-function in centimeters and SDSs, or the timepoint when the function reaches its maximal amplitude, in years.

Change, the calculated growth difference in SDS of the actual QEPS-function between two different timepoints.

	Male					Female				
**Variable**	**Standardized beta (95% CI)**	**p-value**	**R2**	**Partial R2**	**VIF**	**Standardized beta (95% CI)**	**p-value**	**R2**	**Partial R2**	**VIF**
Birth length (cm)						0.103 (0.063 - 0.142)	<.0001	0.06	0.01	1.02
$DiffQE_{max}$ (SDS)	-0.218 (-0.258 - -0.178)	<.0001	0.05	0.05	1.04	-0.246 (-0.285 - -0.206)	<.0001		0.05	1.02
$Change\ QE_{40w\text{-}E99}$ (SDS)	-0.045 (-0.086 - -0.005)	0.026		0.00	1.04					
Beta estimates are standardized both for the dependent and the independent variable.										
