# Supplementary material for: Early life growth is related to pubertal growth and adult height – a QEPS-model analysis
Source: Pediatr Res. 2025 Feb 25;98(4):1339–57. doi: 10.1038/s41390-025-03939-9 (PMC12549337; doi:10.1038/s41390-025-03939-9)
Supplement: Supplementary file 13 — Supplemental Table 3c [file 41390_2025_3939_MOESM13_ESM.pdf]

Supplemental Table 3c: Multivariable total models for $\boldsymbol{Age_{TPHV}}$ (age at peak height velocity of the total growth curve)

Abbreviations: *SDS*, standard deviation scores; *cm*, centimeters

Diff, the calculated differences between the individual's length/height in SDS at the given timepoint and the individual mid-parental height in SDS, i.e. the intrafamilial height difference.

Max, the maximal amplitude of the actual QEPS-function in centimeters and SDSs, or the timepoint when the function reaches its maximal amplitude, in years.

Change, the calculated growth difference in SDS of the actual QEPS-function between two different timepoints.

	Male					Female				
**Variable**	**Standardized beta (95% CI)**	**p-value**	**R2**	**Partial R2**	**VIF**	**Standardized beta (95% CI)**	**p-value**	**R2**	**Partial R2**	**VIF**
Birth weight (grams)						0.070 (0.024 - 0.116)	0.0028	0.09	0.01	1.40
$DiffQE_{max}$ (SDS)	-0.224 (-0.270 - -0.179)	<.0001	0.07	0.07	1.36	-0.294 (-0.334 - -0.255)	<.0001		0.08	1.01
E_{40w} (SDS)						0.053 (0.008 - 0.099)	0.022		0.00	1.38
Q_{E99} (SDS)	-0.064 (-0.109 - -0.018)	0.0061		0.00	1.36					
Beta estimates are standardized both for the dependent and the independent variable.										
