# Supplementary material for: Early life growth is related to pubertal growth and adult height – a QEPS-model analysis
Source: Pediatr Res. 2025 Feb 25;98(4):1339–57. doi: 10.1038/s41390-025-03939-9 (PMC12549337; doi:10.1038/s41390-025-03939-9)
Supplement: Supplementary file 14 — Supplemental Table 3d [file 41390_2025_3939_MOESM14_ESM.pdf]

Supplemental Table 3d: Multivariable total models for ***Adult height SDS***

Abbreviations: *SDS*, standard deviation scores; *cm*, centimeters

Diff, the calculated differences between the individual's length/height in SDS at the given timepoint and the individual mid-parental height in SDS, i.e. the intrafamilial height difference.

Max, the maximal amplitude of the actual QEPS-function in centimeters and SDSs, or the timepoint when the function reaches its maximal amplitude, in years.

Change, the calculated growth difference in SDS of the actual QEPS-function between two different timepoints.

	Male					Female				
Variable	Standardized beta (95% CI)	p-value	R2	Partial R2	VIF	Standardized beta (95% CI)	p-value	R2	Partial R2	VIF
QE_{max} (SDS)	1.129 (1.124 - 1.135)	<.0001	0.99	0.64	1.32	0.839 (0.834 - 0.844)	<.0001	0.98	0.60	1.04
Change E_{E99-P5} (SDS)						0.103 (0.098 - 0.108)	<.0001		0.01	1.04
P_{max} (SDS)	0.590 (0.584 - 0.595)	<.0001		0.25	1.27					
$AgeP_{95}$ (years)	0.325 (0.320 - 0.329)	<.0001		0.10	1.05	0.642 (0.636 - 0.648)	<.0001		0.10	1.45
Change T_{P5-P95} (SDS)						0.596 (0.590 - 0.602)	<.0001		0.27	1.45
Beta estimates are standardized both for the dependent and the independent variable.										
